# Maternally expressed NLRP2 links the subcortical maternal complex (SCMC) to fertility, embryogenesis and epigenetic reprogramming

**DOI:** 10.1038/srep44667

**Published:** 2017-03-20

**Authors:** Sangeetha Mahadevan, Varsha Sathappan, Budi Utama, Isabel Lorenzo, Khalied Kaskar, Ignatia B. Van den Veyver

**Affiliations:** 1 Department of Obstetrics and Gynecology, Baylor College of Medicine, Houston, Texas, 77030, USA; 2 Century Scholars Program, Rice University, Houston, Texas, 77005, USA; 3 Shared Equipment Authority, Rice University, Houston, Texas, 77005, USA; 4 Department of Molecular Human Genetics, Baylor College of Medicine, Houston, Texas, 77030, USA; 5 Interdepartmental Graduate Program in Translational Biology and Molecular Medicine, Baylor College of Medicine, Houston, Texas, 77030, USA; 6 Jan and Duncan Neurological Research Institute, Texas Children’s Hospital, Houston, Texas, 77030, USA

## Abstract

Mammalian parental genomes contribute differently to early embryonic development. Before activation of the zygotic genome, the maternal genome provides all transcripts and proteins required for the transition from a highly specialized oocyte to a pluripotent embryo. Depletion of these maternally-encoded transcripts frequently results in failure of preimplantation embryonic development, but their functions in this process are incompletely understood. We found that female mice lacking NLRP2 are subfertile because of early embryonic loss and the production of fewer offspring that have a wide array of developmental phenotypes and abnormal DNA methylation at imprinted loci. By demonstrating that NLRP2 is a member of the subcortical maternal complex (SCMC), an essential cytoplasmic complex in oocytes and preimplantation embryos with poorly understood function, we identified imprinted postzygotic DNA methylation maintenance, likely by directing subcellular localization of proteins involved in this process, such as DNMT1, as a new crucial role of the SCMC for mammalian reproduction.

Infertility and pregnancy loss are common forms of reproductive failure, with primary and secondary infertility affecting 1.9% and 10.5% of women respectively^1^. Fifteen percent of pregnancies end in a miscarriage and 1% of women who achieve pregnancy will experience recurrent pregnancy loss (RPL), defined as three or more miscarriages^2^,^3^. When a pregnancy fails at the embryonic preimplantation stage, before it is clinically recognized, it presents as primary infertility. Knowledge about causes and treatments for infertility and RPL remain unsatisfactory and it is estimated that for 10–15% of couples with infertility and 50% of women with RPL an etiology cannot be identified. One of the earliest required processes for successful reproduction that has been understudied as a cause of infertility and RPL is the highly dynamic process of transformation from a terminally differentiated oocyte into a pluripotent embryo. Male mammalian germ cells contribute disproportionately to this transformation because prior to zygotic gene activation and initiation of embryonic transcription, the embryo uses maternally-encoded transcripts and proteins for its survival and development^4^. Thus, the key molecular events that take place during early embryogenesis, including transition from meiosis to mitosis, epigenetic reprogramming and a seamless passage from maternal to zygotic transcription, all depend on maternal proteins^5^,^6^,^7^. Characterization of mice with inactivation of the genes that encode these proteins has revealed that pregnancies of mutant females often fail to develop beyond preimplantation embryogenesis. A subset of these “maternal effect genes”, namely *Nlrp5, Tle6, Ooep, Filia (Khdc3*) and *Padi6,* encode proteins of the subcortical maternal complex (SCMC). This large, multimeric, incompletely understood protein complex, is formed in mature oocytes, persists at the periphery of the outermost cells of the cleavage-stage embryo and is absent from the inner cells and areas of cell-cell contact^8^,^9^,^10^,^11^,^12^. Recently, an SCMC containing KHDC3L, NLRP5, OOEP and TLE6 has also been identified in human preimplantation embryos^13^, but the high molecular weights of the human and murine SCMCs indicate that they must contain additional proteins^14^.

In humans, maternal loss of function of some of the genes that encode proteins of the SCMC complex also result in reproductive failure: maternal mutations in *PADI6* and *TLE6* (PREMBL; OMIM# 616814) cause preimplantation embryonic lethality, while women with *KHDC3L* mutations experience a recurrent rare specific form of pregnancy loss, recurrent biparental hydatidiform moles (BiHM) (HYDM2; OMIM# 614293)^15^,^16^,^17^. BiHM pregnancies have abnormal hyperplastic vesicular trophoblast with absent fetal development and loss of DNA methylation at differentially methylated regions (DMRs) of all maternally imprinted genes^17^,^18^. In addition, women with loss of function mutations in the gene encoding NLRP5 (NLR Family, Pyrin Domain-Containing 5), a member of a subclass of NLR family of proteins that is highly expressed in germ cells and preimplantation embryos^19^,^20^, can have recurrent pregnancy loss as well as liveborn children who have abnormal DNA methylation at multiple imprinted loci, indicative of multilocus imprinting disturbance (MLID), and a wide range of clinical phenotypes^21^. These combined observations point to a role for the SCMC in postzygotic maintenance of DNA methylation at imprinted genes^17^,^21^. If confirmed, it would uncover a new class of maternal causes of reproductive failure that range from infertility due to loss of preclinical pregnancies, early recurrent pregnancy loss, to pregnancies that result in offspring with developmental disabilities.

Interestingly, maternal loss of function of two highly homologous genes, *NLRP7* and *NLRP2*, which like *NLRP5*, encode NLR family proteins with predominant expression in germ cells and preimplantation embryos cause relevant phenotypes. Maternal mutations in *NLRP7*, which is absent from rodent genomes, are a more frequent cause of BiHM (HYDM1; OMIM# 231090) than *KHDC3L* mutations. Maternal mutations in *NLRP2,* which is highly homologous to *NLRP7* and immediately adjacent to it on chromosome 19, have been described in one family to result in an MLID that presents as recurrent Beckwith-Wiedemann Syndrome due to loss of DNA methylation at the imprinted *KvDMR1* CpG island (CGI), associated with loss of methylation at the DMR of at least one other imprinted locus, *PEG1*^22^.

The mechanisms by which NLRP2 and NLRP7 impact DNA methylation at imprinted loci are not currently known. Neither is it known whether they are also SCMC members, but if confirmed, it could firmly establish embryonic DNA methylation maintenance as an SCMC function. To examine this in an *in vivo* model, we generated *Nlrp2* null mice on the premise that murine NLRP2 carries out functions that combine those of human NLRP2 and NLRP7, because rodents only have a single *Nlrp2* gene while humans have the highly homologous *NLRP2* and *NLRP7* genes. We observed that loss of maternal NLRP2 results in increased follicular atresia in ovaries of mutant females, subfertility and abnormal cleavage of their embryos when cultured *in vitro. In vivo* developing pregnancies of *Nlrp2-*deficient females produce few offspring that exhibit a spectrum of defects including neonatal death, external structural malformations, and stunted and increased growth with evidence of MLID in embryos and neonatally deceased offspring. We also demonstrated that overexpressed NLRP2 interacts with SCMC proteins, TLE6, OOEP, FILIA and NLRP5 and that subcortical localization of TLE6, a key SCMC protein, was grossly altered in oocytes of *Nlrp2*-null females. Finally, DNMT1, the maintenance DNA methyltransferase, was abnormally localized in oocytes from *Nlrp2*-null females. These results define a new member of the SCMC complex and identify postzygotic maintenance of DNA methylation at imprinted genes, conferred in part by ensuring appropriate localization of DNMT1, as a function of the SCMC that is critical for preimplantation development and successful pregnancy.

## Results

### Loss of NLRP2 affects oocyte morphology and follicle maturation dynamics

**Figure 1:**
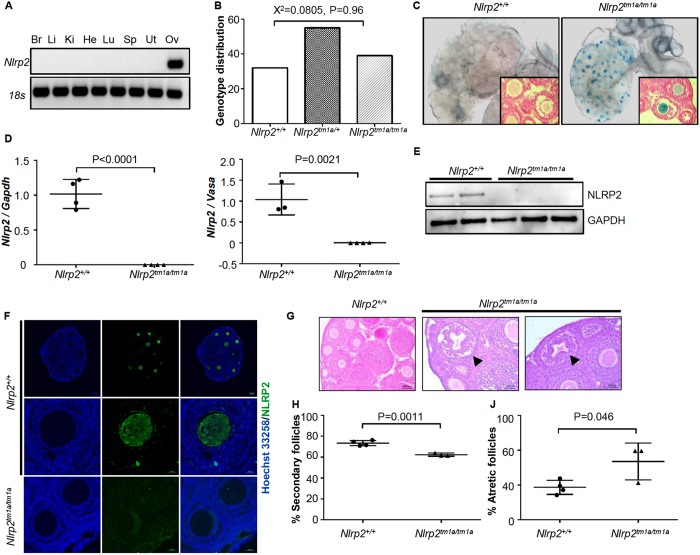
Loss of NLRP2 affects oocyte morphology and follicle maturation dynamics. (**A**) RT-PCR for *Nlrp2* from various tissues shows expression only within the ovary. Br = brain, Li = liver, Ki = kidney, He = heart, Lu = lung, Sp = spleen, Ut = uterus and Ov = ovary. RT-PCR of 18 s serves as a control for RNA integrity. Uncropped, full length gel blots have been provided in Supplementary Figure 1. (**B**) *Nlrp2*^*tm1a/*+^ mice produce offspring in accordance with Mendelian inheritance. (**C**) Whole mount *LacZ* staining followed by clearing of the ovary, oviduct and uterus reveals *LacZ* expression only in ovary, within the follicles. The impression of stage specific NLRP2 expression from *LacZ* staining is likely due to technical reasons and ruled out with immunofluorescence. Inset panels are ovary sections counterstained with nuclear fast red showing *LacZ* expression (blue) only in oocytes, but not in granulosa or theca cells. (**D**) qRT-PCR shows complete absence of *Nlrp2* transcript normalized to *Gapdh* (left) and to *Mvh* (right). (**E**) Western blotting (WB) of lysates of 30 oocytes per lane with anti-NLRP2 shows presence of NLRP2 protein in oocytes of *Nlrp2*^+/+^ females but not*Nlrp2*^*tm1a/tm1a*^. WB with Anti-GAPDH is used as loading control; each lane contains oocytes lysates from an independent 4 week-old superovulated mice. (**F**) Immunofluorescent co-staining of paraffin-embedded ovarian sections from 3 week-old dams with anti-NLRP2 (green; AF488 dye) and Hoechst 33258 (blue) for nuclei, shows that in *Nlrp2*^+/+^ mice, NLRP2 is only found in oocytes (top and middle panels) with a more focused cortical and nucleolar stain observed at higher magnification (middle panels). NLRP2 is not expressed in oocytes of *Nlrp2*^*tm1a/tm1a*^ mice (lower panels) (**G**) Hematoxylin and eosin stain on paraffin-embedded ovaries from *Nlrp2*^+/+^ and *Nlrp2*^*tm1a/tm1a*^ mice reveals grossly abnormal follicles in *Nlrp2*^*tm1a/tm1a*^ ovaries (black arrowheads). Quantification showing fewer secondary (**H**) and atretic (**I**) follicles in *Nlrp2*^*tm1a/tm1a*^ mice. Data are shown as mean ± SEM.

Reverse transcriptase PCR (RT-PCR) on RNA extracted from brain, liver, kidney, heart, lung, spleen, uterus and ovaries of 3-week-old mice revealed that *Nlrp2* expression is restricted to the ovary (Fig. 1A and Supplementary Figure 1). We then generated targeted mice in which *Nlrp2* transcription is abolished and replaced by transcription of a *LacZ* element (Supplementary Figure 2) to generate the *Nlrp2*^*tm1a*^ allele. Seven *Nlrp2*^*tm1a/*+^ breeding pairs had 23 litters with 32 *Nlrp2*^+/+^, 55 *Nlrp2*^*tm1a/*+^ and 39 *Nlrp2*^*tm1a/tm1a*^ offspring (Fig. 1B), which was not significantly different from the expected genotype distributions (Χ^2^ = 0.0805, P = 0.96). *Nlrp2*^*tm1a/*+^ and *Nlrp2*^*tm1a/tm1a*^ mice were viable, without gross morphological or developmental anomalies, grew normally and did not exhibit a decreased lifespan.

β-Gal staining was only visible in the ovaries of *Nlrp2*^*tm1a/*+^ and *Nlrp2*^*tm1a/tm1a*^ females where it is restricted to oocytes (Fig. 1C). qRT-PCR amplification of expressed *Nlrp2* from whole ovaries of 3-week-old mice revealed its complete absence in *Nlrp2*^*tm1a/tm1a*^ mice when normalized to *Gapdh* (P < 0.0001, Fig. 1D, left panel) or to levels of a germ cell specific gene, Mouse Vasa homolog (*Mvh*) (P = 0.0021, Fig. 1D, right panel). The latter indicates that its loss of expression was not secondary to a reduction in the number of germ cells, but that it was missing from existing oocytes. Western blotting on oocyte lysates with a new polyclonal antibody to NLRP2 (DLKNPPLPHFIF), designed to minimize the cross-reactivity with other oocyte-expressed NLRP proteins we observed with commercially available NLRP2 antibodies, showed a specific band at 120 kDa in oocytes from *Nlrp2*^+/+^ females but not in oocytes from *Nlrp2*^*tm1a/tm1a*^ females (Fig. 1E). Immunofluorescence staining with the same antibody on paraffin-embedded ovarian sections from *Nlrp2*^+/+^ females revealed a fluorescent signal restricted to oocytes that was more intense in the cortical and nucleolar regions (Fig. 1F, upper and middle panels); no signal was seen in oocytes of *Nlrp2*^*tm1a/tm1a*^ females (Fig. 1F, lower panels). We next examined ovarian histology at 3-weeks of age and found more unhealthy, degenerating follicles in ovaries of *Nlrp2*^*tm1a/tm1a*^ females, which were also marked by highly irregular oocyte morphology accompanied by increased nuclear pyknosis of granulosa cells (Fig. 1G), significantly fewer secondary follicles (P = 0.0011; Fig. 1H) and more atretic follicles (P = 0.046, Fig. 1I). No significant differences were noted in primary and antral follicles (Supplementary Figure 3). These data indicate that the ovaries of *Nlrp2*^*tm1a/tm1a*^ have subtle alterations in follicle maturation dynamics. This is to our knowledge the first example of a maternal effect mutation that also produces an ovarian phenotype in homozygous mutant females.

### *Nlrp2* ^*tm1a/tm1a*^ mice are subfertile and display a variety of pregnancy outcomes

**Figure 2:**
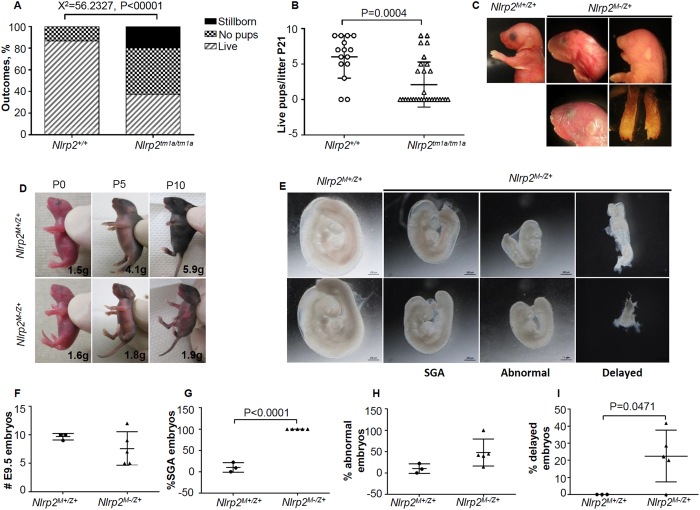
*Nlrp2*^*tm1a/tm1a*^ mice have significantly reduced fertility and a variety of pregnancy outcomes. (**A**) *Nlrp2*^*tm1a/tm1a*^ females (N = 9 mice; 30 litters) have more abnormal reproductive outcomes compared to *Nlrp2*^+/+^ females (N = 10 mice; 15 litters); Outcomes are represented as percentage distribution among all litters. (**B**) The number of pups alive at weaning was significantly reduced in offspring born to *Nlrp2*^*tm1a/tm1a*^ females. (**C**) Craniofacial and skeletal abnormalities noted in *Nlrp2*^*M−/Z*+^ offspring born to *Nlrp2*^*tm1a/tm1a*^ females. (**D**) Severe growth restriction noted in *Nlrp2*^*M−/Z*+^ offspring born to a *Nlrp2*^*tm1a/tm1a*^ female compared to *Nlrp2*^*M*+*/Z*+^ offspring. Body weight was similar at birth but did not increase over time between P0, P5 and P10. (**E**) E9.5 *Nlrp2*^*M−/Z*+^ embryos appear growth restricted compared to *Nlrp2*^*M*+*/Z*+^ embryos; representative examples for otherwise normal but small for gestational age (SGA) embryos, morphologically abnormal embryos, and delayed embryos are shown. (**F**) Number of *Nlrp2*^*M*+*/Z*+^ embryos recovered in each pregnancy at E9.5 from *Nlrp2*^+/+^ females does not differ from *Nlrp2*^*M−/Z*+^ embryos recovered from *Nlrp2*^*tm1a/tm1a*^ females. (**G**) The number of SGA *Nlrp2*^*M−/Z*+^ embryos, which includes small, abnormal and delayed embryos, is significantly higher compared to *Nlrp2*^*M*+*/Z*+^ embryos. (**H**) No difference is noted in the number of abnormal embryos between genotypes. (**I**) There are significantly more severely delayed *Nlrp2*^*M−/Z*+^ embryos compared to *Nlrp2*^*M*+*/Z*+^ embryos. All data are shown as mean ± SEM.

Eight-week-old wild-type and *Nlrp2*^*tm1a/tm1a*^ females were bred with >8-week-old *Nlrp2*^+/+^ males to assess fertility and pregnancy outcomes. We recorded outcomes after 30 observed copulatory vaginal plugs in 9 individual *Nlrp2*^*tm1a/tm1a*^ females and found no offspring born for 13/30 (43%), litters with liveborn offspring for 11/30 (37%), and litters with stillborn pups for 6/30 (20%) observed plugs. Of the 11 litters where liveborn offspring were noted at P0, all pups of 2 litters died within the first few days of life. This was significantly different from outcomes after 15 recorded copulatory vaginal plugs in 10 individual *Nlrp2*^+/+^ mice, of which only 2 (13.3%) did not result in liveborn offspring and 13 (86.7%) were followed by pregnancies that resulted in normal liveborn offspring (Χ^2^ = 56.2327, P < 0.00001, Fig. 2A). Furthermore, the number of pregnancies from which offspring survived to P21 was significantly lower for *Nlrp2*^*tm1a/tm1a*^ mice (P = 0.0004, Fig. 2B).

Given that *Nlrp2*^*tm1a/tm1a*^ females were bred with *Nlrp2*^+/+^ males, all their pregnancies and offspring have a *Nlrp2*^*tm1a/*+^ genotype, which is also found in 50% of pregnancies and offspring from *Nlrp2*^*tm1a/*+^ females bred to *Nlrp2*^+/+^ *or Nlrp2*^*tm1a/*+^ males. While no offspring born to *Nlrp2*^*tm1a/*+^ dams had observable phenotypes, those born to *Nlrp2*^*tm1a/tm1a*^ females had a variable but striking array of developmental abnormalities. This indicates that the lack of maternal *Nlrp2* determines the reproductive outcomes, and henceforth, we will refer to pregnancies, offspring and embryos from *Nlrp2*^*tm1a/tm1a*^ females as *Nlrp2*^*M−/Z*+^ to indicate maternal loss (M−) but zygotic presence (Z+) of the NLRP2 protein. Those from *Nlrp2*^+/+^ females will be referred to as *Nlrp2*^*M*+*/Z*+^ reflecting both maternal (M+) and zygotic presence (Z+) of NLRP2. Some *Nlrp2*^*M−/Z*+^ offspring were found dead on day 1 and had asymmetric developmental abnormalities of limbs, craniofacial abnormalities such as blunted snout, missing or abnormally sized eyes, and micrognathia, and were either larger (*Nlrp2*^*M*+*/Z*+^; PND11 4.920 g ± 0.1158 N = 5, compared to *Nlrp2*^*M*+*/Z*+^; 8.533 ± 0.4177 N = 3, P < 0.0001) or smaller for their age (Fig. 2C and D). A detailed description of specific pregnancy outcomes of the *Nlrp2*^*tm1a/tm1a*^ females is provided in Supplementary Table 1. All null females were isogenic and derived from a single colony and therefore the observation that some females produced no pups at all whereas others had apparently normal pups further highlights the intra- and inter-animal variability due to the NLRP2 mutation. No such anomalous developmental outcome was noted in pups born to *Nlrp2*^*tm1a/*+^ females or *Nlrp2*^*tm1a/tm1a*^males. Considering the combined abnormal follicle maturation and subfertility, we next evaluated if the observed subfertility could be the result of abnormal steroid hormone levels and tested serum levels of anti-Mullerian hormone (AMH), estradiol, progesterone, follicle stimulating hormone (FSH) and luteinizing hormone (LH) in *Nlrp2*^+/+^ and *Nlrp2*^*tm1a/tm1a*^ females, but found no significant differences (Supplementary Figure 4).

To assess if embryonic lethality contributed to the reduced postnatal survival and to examine pregnancies for embryonic structural and growth abnormalities, we performed timed mating of *Nlrp2*^+/+^ or *Nlrp2*^*tm1a/tm1a*^ females with *Nlrp2*^+/+^ males and dissected pregnant females at E9.5. We noted an array of developmental abnormalities in *Nlrp2*^*M−/Z*+^ embryos (Fig. 2E), but observed no statistical difference in the number of embryos recovered from either genotype; 9.6 ± 0.3 embryos/litter for three *Nlrp2*^*M*+*/Z*+^ females and 7.6 ± 1.3 embryos/litter for five *Nlrp2*^*M−/Z*+^ females (P = 0.2911, Fig. 2F). However, all 38 (100%) *Nlrp2*^*M−/Z*+^ embryos were small for gestational age (SGA) of E9.5, compared to only 3 of 29 (10.3%) *Nlrp2*^*M*+*/Z*+^ embryos (P < 0.0001, Fig. 2G). Embryos were defined as ‘abnormal’ if they had absence or abnormal morphology of any of the following features: optic vesicle, otic vesicle, branchial arches 1 and 2 and heart, or if they possessed fewer than 21 somites. Embryos were termed as ‘delayed’ if they presented with features of primitive streak embryos (equivalent to less than ~E6.5) or of ~E8 embryos. By these criteria, 17 of 38 (44.7%) of *Nlrp2*^*M−/Z*+^ embryos were abnormal compared to 3 of 29 (10.3%) of *Nlrp2*^*M*+*/Z*+^ embryos (P = 0.09, Fig. 2H) and 10 of 38 (26.3%) of *Nlrp2*^*M−/Z*+^ embryos were delayed compared to 0 of 29 (0%) of *Nlrp2*^*M*+*/Z*+^ embryos (P = 0.0471, Fig. 2I).

In contrast, when *Nlrp2*^*tm1a/tm1a*^ males were bred with *Nlrp2*^+/+^ females to generate *Nlrp2*^*P−/Z*+^ offspring, outcomes for 43 pups from 7 litters (6.143 ± 0.8 pups/litter) were no different from those of *Nlrp2*^*P*+*/Z*+^ and *Nlrp2*^*M*+*/Z*+^ embryos. Furthermore, there was no increase in adverse outcomes for pregnancies of heterozygous *Nlrp2*^*tm1a/*+^ parents, irrespective of the gender of the *Nlrp2*^*tm1a/*+^ parent. This indicates that, in contrast to the proposed human tissue-specific *NLRP2* imprinting^23^, murine *Nlrp2* is not imprinted. Taken together these data show that *Nlrp2*^*tm1a/tm1a*^ is a maternal effect mutation.

### Maternal loss of NLRP2 results in decreased fertilization rates, abnormal early cleavage and a striking inability to form blastocysts *in vitro*

**Figure 3:**
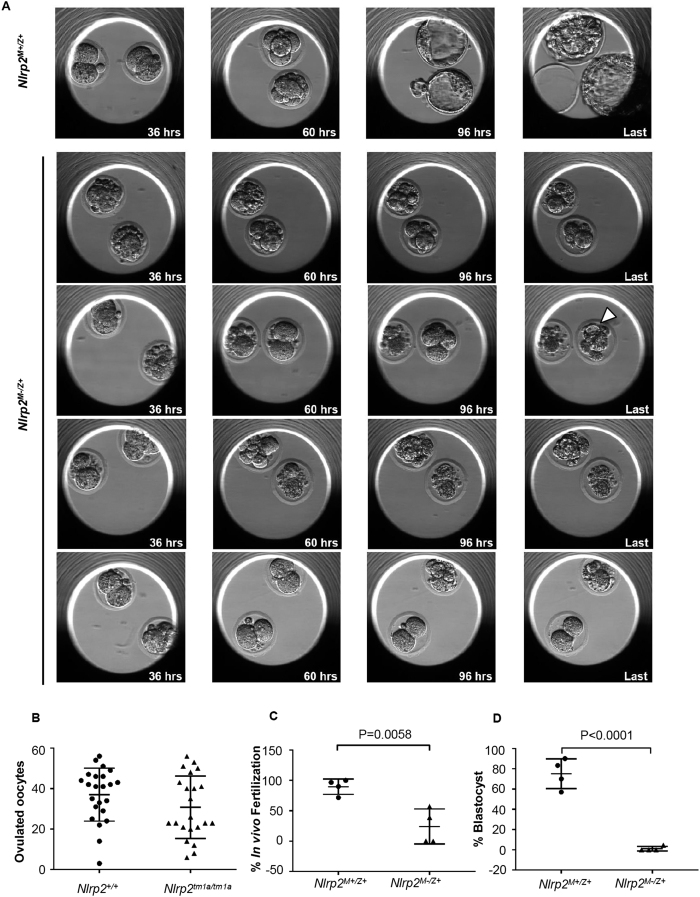
Maternal loss of NLRP2 results in reduced fertilization rates, abnormal early cleavage and inability to form blastocysts in *in vitro* developing embryos. (**A**) Representative still frames at 36, 60, 96 hours and at the end of imaging (~120 hours) from *Nlrp2*^*M*+*Z*+^ and *Nlrp2*^*M−/Z*+^ embryos. Most *Nlrp2*^*M−/Z*+^ embryos arrest at 2-cell stage or degenerate, or rarely, have delayed compaction (white arrowhead) almost 60 hours after it has initiated in *Nlrp2*^*M*+*Z*+^ embryos. (**B**) No difference in the number of ovulated oocytes is noted between genotypes, but (**C**) a significant decrease in the fertilization rates and (**D**) blastocyst formation is noted in *Nlrp2*^*M−/Z*+^ embryos compared to *Nlrp2*^*M*+*Z*+^ embryos. All data are shown as mean ± SEM.

To further examine the role of maternal NLRP2 in early embryo development and characterize the origins of the observed delay in embryogenesis of *Nlrp2*^*M−/Z*+^ embryos at E9.5, we recovered oocytes and zygotes from superovulated dams after mating with proven males for *in vitro* culture with imaging of developing embryos from the 1-cell stage to blastocyst hatching on day 5 using an EmbryoScope time-lapse imaging system. Embryoscopy videos compressed to 3 hours/second are presented in Video S1 (showing two *Nlrp2*^*M*+*/Z*+^ embryos) and Video S2 (showing two *Nlrp2*^*M−/Z*+^ embryos). As shown in representative still frames at 36, 60 and 96 hours and at the end (~120 hours) in Fig. 3A, we found no significant difference between the mean numbers of recovered unfertilized oocytes combined with zygotes per superovulation from 23 *Nlrp2*^+/+^ (37.04 ± 2.730) and 22 *Nlrp2*^*tm1a/tm1a*^ (30.77 ± 3.285) females (Fig. 3B). We next calculated the *in vivo* fertilization rates as the percentage of all recovered zygotes which had two visible pronuclei (2PN), had formed the second polar body, or had formed a cleavage furrow at ~18 hours after presumed fertilization. Of 131 unfertilized oocytes and zygotes recovered from four *Nlrp2*^*tm1a/tm1a*^ females, significantly fewer (24.10 ± 14.38%) were fertilized compared to 105 zygotes recovered from four *Nlrp2*^+/+^ females (89.65 ± 6.305%) (Fig. 3C, P = 0.0058). The percentage of embryos that had formed blastocysts after 5 days of culture was strikingly lower for *Nlrp2*^*M−/Z*+^ embryos (1.125 ± 1.125%) from four *Nlrp2*^*tm1a/tm1a*^ dams compared to *Nlrp2*^*M*+*/Z*+^ embryos (75.15 ± 7.333%) from four *Nlrp2*^+/+^ dams (Fig. 3D, P < 0.0001). The vast majority of fertilized *Nlrp2*^*M−/Z*+^ embryos were arrested at the 2-cell stage or became heavily fragmented and degenerated. The few that progressed to compaction did so with a ~30 hour delay (Fig. 3A, panel marked by white arrowhead).

### NLRP2 interacts with SCMC proteins TLE6, OOEP, FILIA and NLRP5

**Figure 4:**
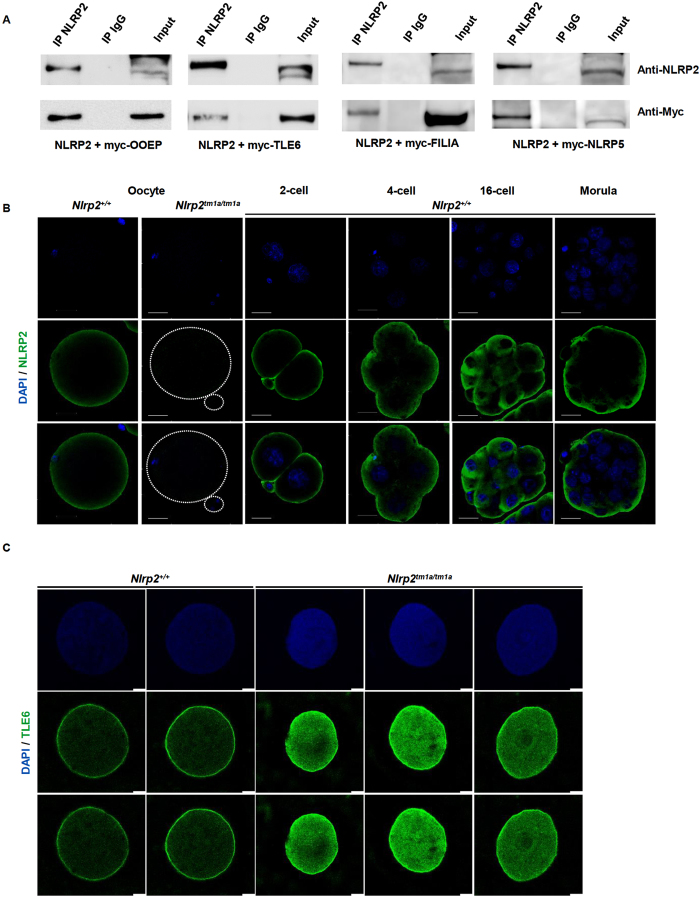
NLRP2 interacts with SCMC components TLE6, OOEP, FILIA and NLRP5. (**A**) NLRP2 was overexpressed with myc-tagged OOEP, TLE6, NLRP5 and FILIA in HEK293T cells for 48 hours, immunoprecipitated with anti-NLRP2 or IgG as negative control and immunoblotted with anti-myc. Top panel shows specificity of anti-NLRP2 IP and bottom panel shows that NLRP2 binds to OOEP, TLE6, FILIA and NLRP5. Uncropped, full length western blots have been provided in Supplementary Figure 5. (**B**) Whole-mount immunofluorescence co-staining with anti-NLRP2 (green) and DAPI (blue) for nuclear staining on Nlrp2^+/+^ oocytes and embryos at 2-, 4-,16-cell and morula stages revealed a predominantly SCMC-like localization for NLRP2. Scale bars represent 200 μm. (**C**) Co-staining with anti-TLE6 (green) and DAPI (blue) of paraffin-embedded oocyte sections reveals a typical cortical stain of TLE6 in oocytes of *Nlrp2*^+/+^ dams but a more intense and diffuse stain in oocytes of *Nlrp2*^*tm1a/tm1a*^ dams. Scale bars represent 10 μm.

The cleavage abnormalities observed in *Nlrp2*^*M−/Z*+^ embryos were similar to those seen in embryos of females with mutations in genes that encode SCMC proteins, which indicated that NLRP2 may be a member of the SCMC. We therefore tested by co-immunoprecipitation of overexpressed proteins in HEK293T cells if NLRP2 interacts with SCMC proteins and found that it binds to TLE6, OOEP, FILIA and NLRP5 (Fig. 4A and Supplementary Figure 5).

Given that SCMC proteins have a characteristic localization in the subcortical region of the oocyte and outermost cells of the preimplantation embryo, we examined expression and subcellular localization of NLRP2 and observed that it is present in oocytes and in preimplantation embryos up to the morula stage. Similar to other SCMC proteins, its localization is predominantly subcortical, but also to a lower extent diffuse through the cytoplasm (Fig. 4B). If maternally contributed NLRP2 is a required protein of the SCMC, the normal subcortical localization of the complex may be altered in oocytes and fertilized *Nlrp2*^*M−/Z*+^ embryos from *Nlrp2*^*tm1a/tm1a*^females. To investigate this but minimize effects on oocytes and embryo cleavage of *in vitro* culture, we dissected the oviducts of superovulated females and performed immunofluorescence staining overnight at 4 °C on paraffin-embedded oocytes with an antibody to TLE6, a representative marker of the SCMC. We found that, compared to oocytes from *Nlrp2*^+/+^ females in which TLE6, consistent with published data, was highly concentrated in a narrow subcortical rim, oocytes from *Nlrp2*^*tm1a/tm1a*^females showed much more intense and diffuse TLE6 staining (Fig. 4C). To address if the discrepant staining pattern observed in SCMC proteins depends on processing procedures^24^, we conducted this staining by a recommended alternate condition that included 1 hour staining at room temperature. Contrary to previous reports we did not observe any difference in TLE6 staining in control oocytes from *Nlrp2*^+/+^ females with different processing conditions. However, staining for 1 hour at room temperature in oocytes from *Nlrp2*^*tm1a/tm1a*^females showed much weaker TLE6 staining (Supplementary Figure 6). This observation contrasts the findings when the staining is carried out overnight at 4 °C and are more consistent with other reports where knockout of one of the SCMC components results in loss of characteristic expression of other SCMC proteins. These findings also suggest that processing conditions for fluorescent staining of sub-cortical proteins can influence observed localization.

### Abnormal DNA methylation at imprinted loci in stillborn offspring and embryos of *Nlrp2* ^*tm1a/tm1a*^females

**Figure 5:**
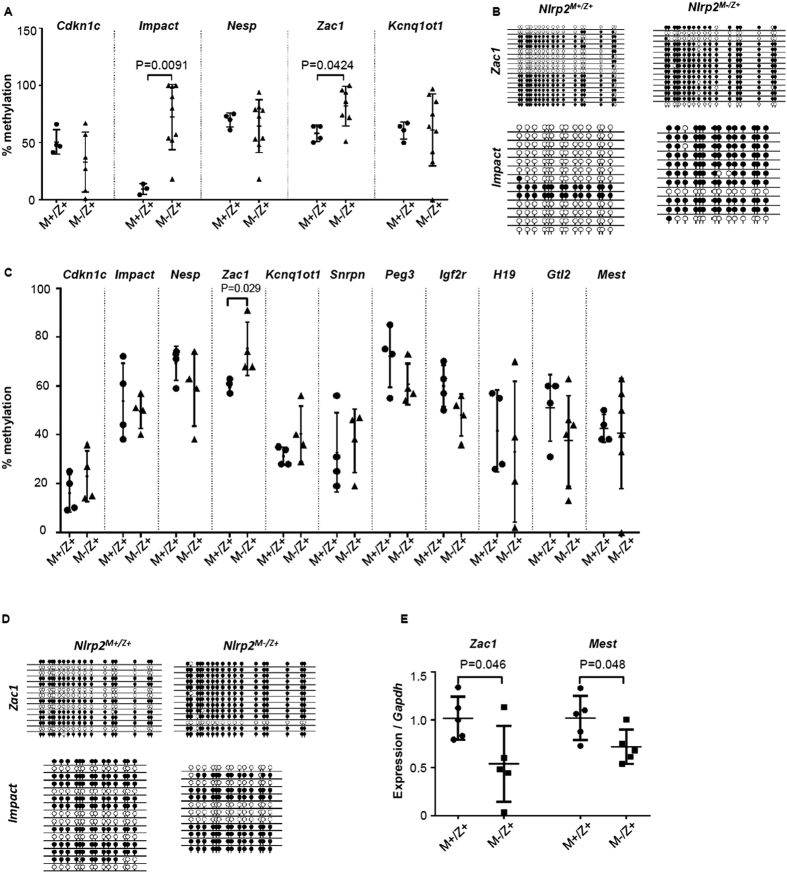
Abnormal DNA methylation at imprinted loci in stillborn offspring and embryos of *Nlrp2*^*tm1a/tm1a*^ females. (**A**) Bisulfite cloning and sequencing was carried out on 16–32 clones at maternally expressed gene *Cdkn1c* and maternally imprinted genes *Impact, Nesp, Zac1*, and *Kcnq1ot1* from *Nlrp2*^*M−/Z*+^ control and *Nlrp2*^*M*+*/Z*+^ stillborn pups. Data is represented as scatterplots of % methylation; where each dot represents methylation at the tested locus per sample. (**B**) Representative scaled lollipop diagrams from *Nlrp2*^*M−/Z*+^ and *Nlrp2*^*M*+*/Z*+^ stillborn pup cloning at the *Zac1* and *Impact* loci are shown. Filled circles represent methylated CGs and empty circles represent unmethylated CGs, each row represents a sequenced clone. (**C**) Bisulfite cloning and sequencing was carried out on 16–32 clones of DNA extracted from *Nlrp2*^*M−/Z*+^ and *Nlrp2*^*M*+*/Z*+^ embryos at *Cdkn1c, Impact, Nesp, Zac1*, and *Kcnq1ot1, Snrpn, Peg3, Igf2r, H19, Gtl2 and Mest*. (**D**) Representative scaled lollipop diagrams from *Nlrp2*^*M−/Z*+^ and *Nlrp2*^*M*+*/Z*+^ E9.5 embryo cloning at the *Zac1* and *Impact* loci are shown. Filled circles represent methylated CGs and empty circles represent unmethylated CGs, each row represents a sequenced clone. (**E**) qRT-PCR of *Nlrp2*^*M−/Z*+^ and *Nlrp2*^*M*+*/Z*+^ E9.5 embryos reveals decrease in *Zac1* and *Mest* gene expression.

Considering that in humans, maternal effect mutations in *NLRP2* and other members of the NLRP family (*NLRP7* and *NLRP5*), as well as *KHDC3L* affect imprinted DNA methylation in trophoblast or offspring^17^,^18^,^21^, we next investigated if the embryological cleavage defects, embryonic growth restriction, abnormal development and stillbirth of offspring from *Nlrp2*^*tm1a/tm1a*^females could also be a consequence of abnormal imprinted DNA methylation. We analyzed DNA methylation at DMRs of maternally imprinted genes *Impact, Nesp, Zac1*, and *Kcnq1ot1* and maternally expressed *Cdkn1c* on bisulfite-converted genomic DNA extracted from stillborn pups. In addition to the above listed genes, we also analyzed *Snrpn, Peg3, Igf2r* and paternally imprinted genes *H19* and *Gtl2* on bisulfite-converted genomic DNA extracted from E9.5 embryos. We found a non-significant trend for a mixed pattern of gain and loss of methylation at *Nesp* and *Kcnq1ot1* and for loss of methylation at *Cdkn1c* in stillborn pups. However, we observed a significant gain of methylation at *Zac1* (P = 0.0424) and *Impact* (P = 0.0091) in stillborn *Nlrp2*^*M−/Z*+^ pups (Fig. 5A and B), together with a more selective methylation gain at *Zac1* in *Nlrp2*^*M−/Z*+^ embryos at E9.5 (P = 0.029, Fig. 5C and D). We did not observe an increase in methylation at *Mest*, however *Nlrp2*^*M−/Z*+^ embryos exhibit significantly greater variability in levels of methylation compared to *Nlrp2*^*M*+*/Z*+^ control embryos (F statistic for variance P = 0.027). Supplementary Table 2 contains specific methylation levels for all the analyzed pups and embryos. To assess if the alterations in DNA methylation were associated with abnormal expression of tested imprinted genes, we analyzed expression of maternally imprinted genes *Impact, Mest, Grb10, Snrpn, Peg3, Cdkn1c* and *Igf2r* and of the paternally imprinted gene *H19*. We found that the gain in methylation in *Zac1* was accompanied by decreased expression of *Zac1* (P = 0.0459) and also noted that *Mest*, which had more variable methylation, had lower expression (P = 0.048) (Fig. 5E). The observation that maternal deficiency of NLRP2 results in mixed losses and gains of methylation indicates a possible stochastic feature to its function. Intriguingly, we also found that while methylation of the paternally imprinted genes *H19* and *Gtl2* were not significantly different, they were more variable in E9.5 *Nlrp2*^*M−/Z*+^ embryos compared to *Nlrp2*^*M*+*/Z*+^ control embryos (Fig. 5C), indicating that altered embryonic DNA methylation subsequent to absence of maternally contributed NLRP2 is not restricted to maternally imprinted genes.

**Figure 6:**
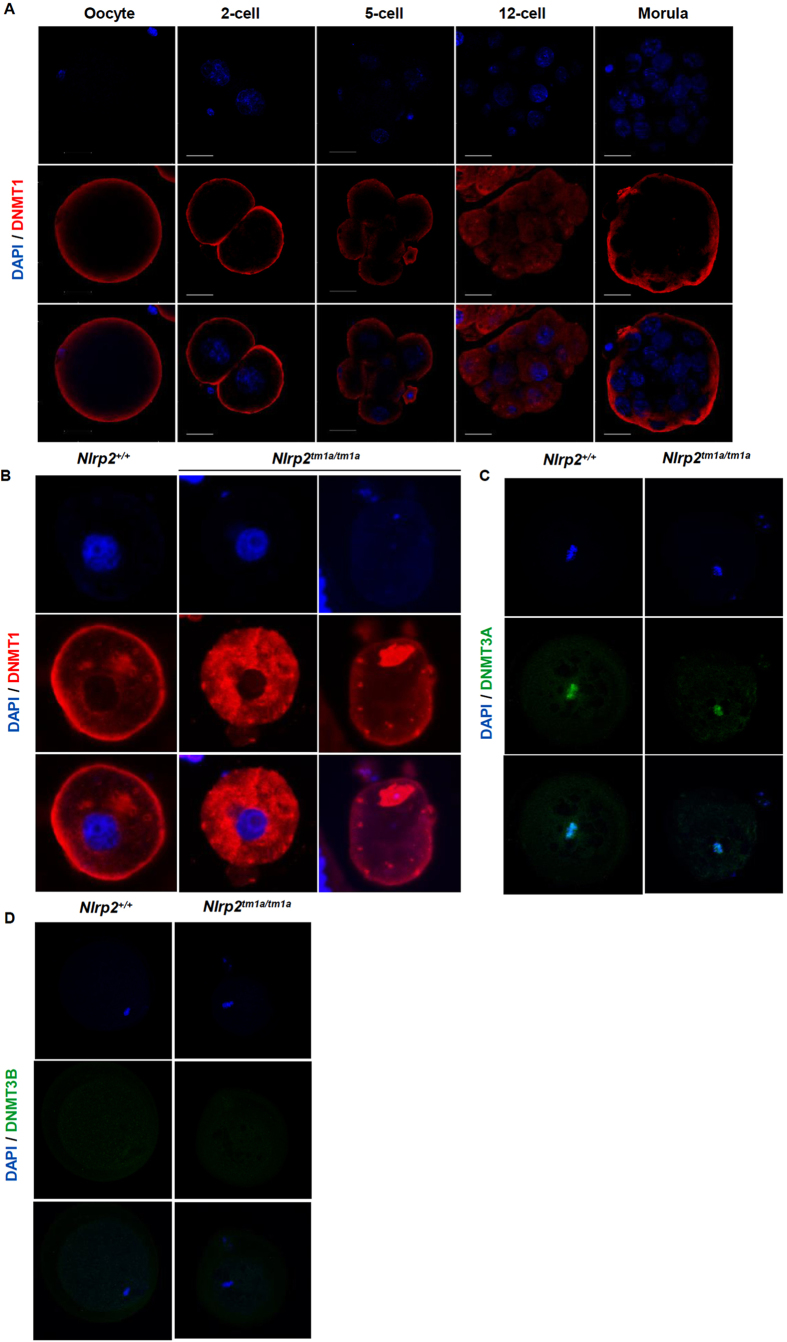
DNMT1 reveals a SCMC-like cortical localization with aberrant localization in *Nlrp2*^*tm1a/tm1a*^ derived oocytes. (**A**) Whole mount immunofluorescence co-staining with anti-DNMT1 and DAPI on the same *Nlrp2*^*M*+*/Z*+^ embryos used to stain NLRP2 reveals a SCMC-like cortical localization of DNMT1 with a diffuse nuclear stain in later stage embryos. Scale bar represented is 200 μm. (**B**) DNMT1 staining in paraffin embedded oocyte sections reveals a less cortical, more cytoplasmic intense stain with nucleolar focus in oocytes from *Nlrp2*^*tm1a/tm1a*^ dams compared to oocytes from *Nlrp2*^+/+^ dams. (**C**) Whole mount immunofluorescence for DNMT3A in unfertilized control oocytes reveals a characteristic metaphase associated localization and no difference is noted in oocytes derived from *Nlrp2*^*tm1a/tm1a*^dams. (**D**) Whole mount immunofluorescence in unfertilized oocytes reveals that as expected, DNMT3B is not yet expressed in unfertilized control oocytes and no expression is noted in oocytes derived from *Nlrp2*^*tm1a/tm1a*^dams.

To investigate the mechanisms by which lack of a maternally provided protein could impact methylation at both maternally and paternally imprinted genes, we examined the subcellular localization of DNA methyltransferase 1 (DNMT1) in oocytes and embryos from *Nlrp2*^+/+^ and *Nlrp2*^*tm1a/tm1a*^females. We found that in oocytes and *Nlrp2*^*M*+*/Z*+^ preimplantation embryos of *Nlrp2*^+/+^ females, expression and localization of DNMT1 was similar to that of NLRP2 and other SCMC proteins, with a striking cortical concentration of DNMT1 along with a weaker, more diffuse cytoplasmic signal (Fig. 6A). Because preimplantation development of *Nlrp2*^*M−/Z*+^ embryos is severely constrained, we investigated the expression and localization of DNMT1 in ovulated oocytes of *Nlrp2*^*tm1a/tm1a*^ females by immunofluorescence staining on paraffin embedded postovulatory oviducts. We found that like TLE6, DNMT1 had a stronger and more diffuse localization in fertilized oocytes of *Nlrp2*^*tm1a/tm1a*^ females compared to those of *Nlrp2*^+/+^ females, potentially suggesting that the disruption of the SCMC affects proper localization of DNMT1. Additionally, a denser concentration of DNMT1 was noted in the nucleoli of oocytes from *Nlrp2*^*tm1a/tm1a*^ females (Fig. 6B). We then assessed expression and localization of DNMT3A and DNMT3B in control and targeted oocytes as previously described^25^. As expected from published reports, DNMT3A is associated with metaphase chromosomes in oocytes of *Nlrp2*^+/+^ females and no difference is noted in oocytes of *Nlrp2*^*tm1a/tm1a*^ females (Fig. 6C). DNMT3B is not expressed in oocytes and therefore no expression is noted in either control oocytes or those derived from *Nlrp2*^*tm1a/tm1a*^females (Fig. 6D).

## Discussion

At the onset of this study, little was known about the function of *NLRP2*, except that it is abundant in early embryos, that in one family its maternal loss in humans causes an imprinting disorder in offspring^22^ and that it is highly homologous to *NLRP7*, maternal mutations of which cause recurrent and abnormal pregnancies with multilocus imprinting defects. Considering that *NLRP7* is absent from the mouse genome, we speculated that the product of the murine *Nlrp2* gene might carry out functions that combine those of human NLRP2 and NLRP7, and consequently that mice lacking *Nlrp2* would exhibit an inability to establish healthy pregnancies. We indeed found that *Nlrp2*^*tm1a/tm1a*^ male and female mice were indistinguishable from their wild type and heterozygous littermates for growth, mating behavior and survival. While *Nlrp2*-deficient males had normal fertility, females had a wide range of reproductive outcomes. There were fewer and smaller litters, increased neonatal death and a mixture of offspring with abnormal growth and multiple developmental defects. Despite the lower numbers of offspring observed after full-term *in vivo* pregnancies, we found that although most embryos were small or abnormally developed at E9.5, the number of embryos was not different from *Nlrp2*^+/+^ mice, suggesting that resorption occurred later. In contrast, *Nlrp2*^*M−/Z*+^ embryos that were flushed from the oviduct after mating of superovulated females and then cultured *in vitro* became heavily fragmented and did not form blastocysts. This surprising finding suggests that in mice the embryonic phenotype is exacerbated by hormonal ovulation induction, embryo culture or their combination.

These studies are reminiscent of the findings from *in vivo* siRNA knockdown of *Nlrp2* which resulted in mostly fragmented and degenerated embryos that were unable to reach the blastocyst stage^25^. In humans, the primary presentation of *NLRP2* maternal effect mutations, is a Beckwith-Wiedemann phenotype in offspring, whereas mutations in *NLRP7* cause recurrent biparental hydatidiform moles, a placental developmental phenotype. To address if *Nlrp2* maternal effect mutations are also associated with placental abnormalities, it will be important to conduct detailed investigations of placental morphology and cell-type specific imprinting disturbances in sorted placental cell populations from pregnancies of *Nlrp2*^*tm1a/tm1a*^ females.

The inability of *Nlrp2*^*M−/Z*+^ embryos to cleave normally *in vitro* is very similar to what is found in mice with mutations in genes encoding members of the SCMC, thus we were not surprised to find that NLRP2 binds to SCMC proteins, localizes in a pattern that overlaps significantly with the subcortical localization of the SCMC in oocytes and preimplantation embryos and that its loss alters the distribution of TLE6, a core SCMC protein. Intriguingly, in contrast to females with inactivation of *Nlrp5, Tle6* or *Ooep*, who never achieve a successful pregnancy, 37% of embryos and offspring of *Nlrp2*^*tm1a/tm1a*^ females were able to develop post-implantation and a subset of these were apparently normal at birth. This suggests that although NLRP2 is a member of the SCMC, either the integrity of the SCMC is not fully dependent on NLRP2, or in some cases development can proceed even in the absence of an intact SCMC. Further research will be needed to differentiate between these two possibilities.

The variable phenotypes of *Nlrp2*^*M−/Z*+^ pregnancies and offspring indicate that the consequences of maternal loss of *Nlrp2* on reproductive outcomes are stochastic, which suggests that an epigenetic mechanism is involved. We therefore examined if murine *Nlrp2,* similar to human *NLRP2* and *NLRP7*, causes loss of DNA methylation at DMRs of imprinted genes. We found that, unlike the complete loss of DNA methylation at maternally imprinted genes noted in the pregnancies of women with *NLRP7* loss of function mutations, *Nlrp2*^*M−/Z*+^ offspring and embryos exhibited a wide range of DNA methylation gains and losses. The increase in DNA methylation and reduced mRNA from the *Zac1* locus in *Nlrp2*^*M−/Z*+^ offspring is intriguing, because the developmental abnormalities in the paternal knockout of *Zac1* are strikingly similar to those in *Nlrp2*^*M−/Z*+^ offspring^26^ and we speculate that reduced expression of *Zac1* causes some of the phenotypes we observed, but aberrant gains and losses in DNA methylation causing altered expression at other imprinted genes may also contribute. We next examined if the altered DNA methylation at imprinted loci could be due to erroneous expression of DNA methyltransferases that are active during the 1 to 8 cell stage. The somatic variant of DNMT1s is believed to contribute, at least in part, to maintenance of DNA methylation at imprinted loci during preimplantation embryogenesis, and especially prior to nuclear shuttling of the oocyte provided DNMT1o^27^,^28^,^29^,^30^. The finding that DNMT1 is more highly expressed and mislocalized in *Nlrp2*^*M−/Z*+^ embryos is noteworthy and likely contributes to the aberrant gain in DNA methylation. However, the H-300 DNMT1 antibody we used detects both oocyte and somatic DNMT1 variants since the epitope is at the C-terminal region of the protein. It is difficult to comment, therefore, on whether it is the somatic or oocyte variant that appears upregulated or mislocalized, but this will be the subject of our future studies. The staining patterns of DNMT3A and DNMT3B have been extensively studied in murine oocytes and preimplantation embryos. DNMT3A is highly expressed in the nucleus of fully grown oocytes and associates with metaphase chromosomes in MII oocytes^27^. Following fertilization, DNMT3A remains nuclear and its staining intensity is lower by blastocyst stage. In contrast, DNMT3B is not expressed in oocytes or in preimplantation embryos until the 4-cell stage. Our whole mount immunofluorescence in oocytes confirmed DNMT3A’s association with metaphase chromosomes and absence of DNMT3B in oocytes from wild-type and *Nlrp2*^*tm1a/tm1a*^ dams. Due to the difficulties in culturing embryos from *Nlrp2*^*tm1a/tm1a*^ dams beyond the two-cell stage, we restricted our current analysis to oocytes, but plan to examine in future experiments if expression in developing embryos from *Nlrp2*^*tm1a/tm1a*^ dams is abnormal. Understanding these differences will provide greater insight into the specific mechanisms by which SCMC proteins affect postzygotic imprinted DNA methylation. Interestingly, embryos obtained from oocytes that lack maternal KDM1A, another epigenetic factor that contributes to imprinting, also exhibit hypermethylation at *Zac1* and *Impact*^31^.

One of our objectives was to understand if loss of maternal NLRP2 compromised fertility and impacted embryonic development. We found a wide range of phenotypes of which the mid-gestation embryos and pups that developed to term probably represent the milder spectrum and associated imprinting defects caused by maternal loss of NLRP2. It will be important to assess in the future, imprinting in oocytes and preimplantation *Nlrp2*^*M−/Z*+^ embryos. These studies can clarify if murine NLRP2 functions more like human NLRP7 which appears to be necessary for imprint acquisition^18^,^32^,^33^, or like human NLRP5^21^ which appears to function during imprint maintenance. Because murine NLRP2 likely carries out the functions of human NLRP2 and NLRP7, establishing whether murine NLRP2 functions in imprint acquisition or maintenance may also shed light on the function of human NLRP7.

An important new observation is that mouse NLRP2 interacts with proteins of the SCMC. This is complemented with our unpublished data that human NLRP7 and NLRP2 interact with SCMC components KHDC3L, TLE6 and OOEP. Furthermore, the localization of murine NLRP2 also resembles that of NLRP7’s cortical localization with decreasing intensity towards regions of cell to cell contact^34^. Collectively, this indicates that NLRP2 and NLRP7 are themselves SCMC members. Thus, our data on altered DNA methylation, together with the abnormal DNA methylation noted in pregnancies of women with mutations in *NLRP7* and *KHDC3L*, strongly supports that one of the functions of the SCMC is postzygotic maintenance of DNA methylation. This is surprising given the subcortical cytoplasmic localization of SCMC proteins, since DNA methylation is a nuclear process. Considering that certain SCMC mutants have decreased maternal stores of RNA and protein, one could speculate that in addition to the observed changes in DNMT1, stores of proteins such as PGC7, KAP1 and ZFP57, which all function to maintain DNA methylation in the preimplantation embryo^35^,^36^,^37^ may be mislocalized, depleted or perhaps not appropriately degraded when the embryonic genome becomes activated. While the striking cortical localization of DNMT1 suggests that it could be bound to the SCMC, it could also be that reserves of critical maternal proteins are localized at the cortical rim of the developing embryo, such that destabilization of the SCMC indirectly impacts nearby DNMT1, but other processes may also contribute. For example, a proposed major function of the SCMC is to ensure faithful maintenance of ploidy and symmetric cell division^11^. Thus, abnormal cleavage might affect the cell cycle of individual cells within a developing embryo, further impacting appropriate localization of DNA methyltransferases. A final scenario may simply be that SCMC-mutant oocytes and the embryos derived from them lack the appropriate stoichiometry of maternal proteins such that abnormal DNA methylation is a bystander effect to a generalized state of crisis in the embryo. Future studies will be needed to differentiate between these possible mechanisms.

In conclusion, we show that in mice, maternal loss of NLRP2 results in significantly compromised fertility and defective embryogenesis with variably penetrant multi-locus imprinting disturbances. This supports the observation that embryos deficient for at least one of the maternally contributed SCMC proteins can have aberrant DNA methylation, either directly or as a bystander to the generalized developmental disruption of the embryos. This information also suggests that abnormal DNA methylation should be investigated in pregnancies of other SCMC mutants. The observed reproductive outcomes and impact on DNA methylation also indicate that there is both functional overlap and divergence between the murine *Nlrp2* and human *NLRP2* and *NLRP7* genes. The findings from this study further imply that women with unexplained primary infertility due to preclinical pregnancy loss or with early arrest of all embryonic development during IVF and pervasive failure of development following IVF may harbor mutations in this and other genes that encode SCMC proteins or in genes that encode other oocyte-expressed proteins that are important for reprogramming of DNA methylation. While the mutational frequency of SCMC genes in unexplained infertility and women with enduring failure of development following IVF remains to be determined, it is an important cause to recognize, as it would not be treatable by *in vitro* fertilization, unless donor oocytes are used. Finally, this work combined with prior studies and clinical observations define maternal effect gene mutations as a new category of genetic defects that decrease reproductive fitness.

## Materials and Methods

The study protocol (AN-2035) was approved by the Institutional Animal Care and Use Committee (IACUC) at Baylor College of Medicine (BCM). All experiments were conducted according to institutional and governmental regulations concerning the ethical use of animals. All animal facilities are approved by the Association for Assessment and Accreditation for Laboratory Animal Care International (AAALAC).

### Assessment of fertility

7–8 week old female *Nlrp2*^+/+^ and *Nlrp2*^*tm1a/tm1a*^ mice were housed with >8 week old *Nlrp2*^+/+^ male mice between 1600–1800 hours and checked for the presence of vaginal plug the following morning. If a vaginal plug was observed, the male was removed from the cage and females monitored closely beginning at E18.5, for parturition. At birth, the gestational length, number of offspring born and gender distributions were noted. In instances where live, morphologically abnormal offspring were found, body weights were recorded and photographs were taken. In instances where offspring were found deceased, photographs were taken and tissues preserved for subsequent analysis. If the female did not deliver beyond 25 days post coitus, the pregnancy outcome was recorded as “no outcome”.

### Western blotting

Three commercially available NLRP2 antibodies were tested in Western Blotting and Immunofluorescence (IF) /Immunohistochemistry (IHC) applications. We tested Anti-NLRP2 antibody produced in rabbit (Sigma-Aldrich, St. Louis, MO; Cat #SAB1411064), Anti-NALP2 antibody produced in rabbit (Sigma-Aldrich, St. Louis, MO; Cat #SAB3500325) and NLRP2 MaxPab mouse polyclonal antibody (B01) (Abnova, Taipei; Cat # H00055655-B01) on either whole ovary protein lysates for western blotting or paraffin embedded ovary sections for IHC.

Due to the non-specificity of commercially available mouse reactive NLRP2 antibodies, we generated a goat polyclonal antibody against the C terminal DLKNPPLPHFIF sequence (Bethyl Laboratories, Inc.). Western blotting on oocyte lysates were performed as follows. 30 denuded oocytes were denatured at 100 °C for 10 minutes in 4X Bolt LDS Sample Buffer (Life Technologies, Cat #B0007) and 10X Bolt Sample Reducing Agent (Life Technologies, Cat #B0004). Oocyte lysates were run on Bolt 4–12% Bis-Tris Plus Gels (Life Technologies, Cat #NW04120BOX) and transferred to a nitrocellulose membrane. Membranes were blocked in 5% blotting grade blocker (Bio-Rad, Cat # 1706404) in PBS, 0.1% Tween-20. Primary antibodies were diluted in the blocking solution at 1:250. HRP conjugated Donkey anti goat IgG HRP (Santa Cruz Biotechnology, Cat # sc-2020) secondary antibody was used. Substrates were detected using the SuperSignal West Femto chemiluminescent substrates (Thermo Fisher Scientific, Cat #34077, 34094). All western blots were carried out on a minimum of 3 per genotype and repeated thrice.

### Immunofluorescence

Immunofluorescence was carried out on 4 μM sections from paraffin embedded ovaries and ampulla collected from superovulated females. The tissues were processed as listed under histological studies. Sections were de-paraffinized and dehydrated following which a Citrate based antigen retrieval solution (pH6) was used to recover the epitopes. Sections were blocked in 5% normal donkey serum in 1X PBS, 0.1% Tween-20 and the same blocking solution was used to dilute primary and secondary antibodies. Primary antibodies were incubated in humidified conditions overnight at 4 °C. The following antibodies were used at the indicated dilutions: Anti-NLRP2 (Bethyl, custom generated polyclonal, 1:50), anti-TLE6 (Santa Cruz Biotechnology, Cat #sc-162320, 1:50). Secondary antibodies used were Alexa Fluor 594 Donkey Anti-Rabbit IgG (Thermo Fisher Scientific, Cat # A-21207) and Alexa Fluor 488 Rabbit anti-Goat IgG (Thermo Fisher Scientific, Cat #A-21222, 1:500). To assess if processing conditions alter the observed TLE6 staining pattern, the primary antibody incubation was also carried out at room temperature for 1 hour. The remainder of the protocol was unchanged. NucBlue^®^ Fixed Cell ReadyProbes^®^ Reagent (Thermo Fisher Scientific, Cat #R37606) was used to stain nuclei. ProLong^®^ Gold Antifade Mountant (Thermo Fisher Scientific, Cat # P36930) was used to mount the slides. Slides were images using a Zeiss LSM 880 with Airyscan Confocal Microscope at the Neurological Research Institute Microscopy core. Whole mount immunofluorescence was performed as follows: P28 mice were superovulated as described earlier and mated with *Nlrp2*^+/+^ males. The following morning, E0.5 zygotes were collected form the ampulla, denuded and cultured as described under embryoscope studies. At 24 hour intervals, zona containing embryos were rinsed in M2 media and fixed in 4% PFA for 20 minutes at room temperature. Embryos were then permeabilized with 1% triton X-100 in 1X PBS prior to being blocked in 1% NDS (normal donkey serum) in the permeabilization solution. Antibodies were diluted in the blocking solution as follows: Anti-NLRP2 (primary 1:250 overnight at 4 °C, secondary Alexa Fluor 488 1:1000 1 hour at room temperature), Anti-DNMT1 (Santa Cruz Biotechnology, Cat #sc-20701 primary 1:250 overnight at 4 °C, secondary Alexa Fluor 594 1:1000 1 hour at room temperature). For staining with anti-DNMT3A (Novus Biologicals, Cat #NB120-13888SS) and anti-DNMT3B (Novus Biologicals, Cat # NB100-56514SS), the following conditions were used: Superovulated oocytes were fixed for 30 minutes in 3.7% paraformaldehyde on ice. Following washes in 1X PBS, oocytes were treated with a pre-treatment buffer (2% triton X-100 in 1X PBS) for 30 minutes. Oocytes were then transferred to primary antibody solutions (1:500 diluted in 0.1% triton X-100 in 1X PBS) overnight at 4 °C. Following washes with 1X PBS, oocytes were transferred to secondary antibody solutions (Alexa Fluor 488 1:2000 diluted in 0.1% triton X-100) for 30 minutes at room temperature. Oocytes and embryos were mounted on glass slides and imaged as described above. All laser intensities and saturations were maintained consistently during imaging. Raw images were deconvoluted using AutoQuant X3 and further processed using Imaris version 8.2.

### Embryo culture

Superovulated oocytes were rinsed in Quinns Advantage Medium with HEPES (Origio, Cat # ART-1023) and then loaded onto EmbryoSlide culture dishes (Unisense, Fertilitech Cat # FT-S-ES-D) with 25 μL of Quinns Advantage Protein Plus Cleavage Medium (Origio, Cat # ART-1526) and overlaid with mineral oil (LifeGlobal, Cat # LGUA-500). Approximately 48 hours later, embryos were transferred to Quinns Advantage Protein Plus Blastocyst Medium (Origio, Cat # ART-1529). Embryos were cultured at 37 °C with 6% CO_2_ and 6% O_2_ in a humidity-free environment for a total of 5 days. The EmbryoScope time lapse system (Vitrolife) captured images every 10 minutes for the entire duration of culture and videos generated from these images were compressed to 3 hours/second. Fertilization was confirmed by the presence of 2 pronuclei, second polar body or evidence of the formation of the cleavage furrow. The EmbryoScope imaging system used was from the research laboratory of the Family Fertility Center at the Texas Children’s Hospital Pavilion for Women.

### NLRP2 interactor studies

HEK293T cells were cultured in Dulbecco’s Modified Eagle Medium (Lonza Cat #12-604 F/12) with 10% Fetal Bovine Serum and 1X antibiotics at 37 °C with 5% CO_2_. The following plasmids were transfected into HEK293T cells using the PolyJet™ *In Vitro* DNA Tranfection Reagent (SignaGen Laboratories, Cat #SL100688). N´FLAG-NLRP2 (Genecopoeia, Cat #EX-Mm25920-M11), C´Myc-FILIA (Genecopoeia, Cat # EX-Mm08903-M09), C´Myc-DDK-TLE6 (Origene, Cat #MR208164), C´Myc-DDK-OOEP (Origene, Cat #MR201316) and C´Myc-NLRP5 (Genecopoeia, Cat # EX-Mm21358-M09). Because the co-transfection efficiency of NLRP2 and FILIA/NLRP5 was low, we used Lipofectamine 2000 (Thermo Fisher Scientific, Cat # 11668027). The remainder of the steps were processed the same, regardless of transfection agent. Plasmids were overexpressed in HEK293T cells for 48 hours, following which protein was isolated using a NP-40 based lysis buffer. Protein A Dynabeads (Thermo Fisher Scientific, Cat # 10002D) were conjugated with 5 μg of anti-NLRP2 antibody and used to immunoprecipitate 300 μg of protein. IgG-conjugated dynabeads were used for pre-clearing the lysates and for serving as a negative control. 10% of protein was reserved as input. Immunoprecipitated products and inputs were analyzed by western blotting as described in an earlier section. Interacting proteins were detected using an anti-Myc antibody (Novus Biologicals, Cat #NB600-302).

### Bisulfite sequencing

Genomic DNA was isolated from the facial regions of the deceased pups born to *Nlrp2*^*tm1a/tm1a*^ females. All pups had a genotype of *Nlrp2*^*tm1a*/+^. Control pups also had an *Nlrp2*^+/+^ or *Nlrp2*^*tm1a*/+^ genotype but were born to *Nlrp2*^*tm1a/*+^ females. 3–4 pups from 3 *Nlrp2*^+/+^ and *Nlrp2*^*tm1a/*+^ females were used as controls. Six to nine pups from 3 *Nlrp2*^*tm1a/tm1a*^ females were used for analysis. E9.5 embryos were used as a whole for DNA isolation. DNA was isolated using the Gentra Puregene Blood Kit (Qiagen, Cat # 158445) and 200 ng–1 μg of DNA was bisulfite converted using the EZ DNA Methylation-Direct Kit (Zymo Research, Cat # D5020). Six embryos from 2 *Nlrp2*^+/+^ females and 8 embryos from 2 *Nlrp2*^*tm1a/tm1a*^ females were used for analysis. Depending on the developmental competence of the E9.5 embryos, we recovered 50 ng to several micrograms on DNA. In instances where the amount of DNA was less than 200 ng, bisulfite conversion was carried out on as much DNA was available. Primers used to amplify imprinted loci are provided in Supplementary Table 3. PCR products were cloned into the PCR4 TOPO TA cloning vector (Life Technologies, Cat # K4575-02). sSixteen to thirty-two clones were selected following blue white screening for sequencing using the M13 reverse primer (GeneWiz, New Jersey). CpG viewer provided by the Leeds Institute of Molecular Medicine was used to generate scaled lollipop diagrams (Carr IM, Valleley EMA, Cordery SF, Markham AF & Bonthron DT (2007). Sequence analysis and editing for bisulphite genomic sequencing projects. Nucleic Acids Res., 35:e79). An in-house script was written in Python to quantify percentage of methylated versus unmethylated CGs in the sequences. CpG viewer was used to align chromatograms with the bisulfite converted reference sequence. CpG Viewer was then used to export files as lollipop diagrams (as shown in Fig. 5 and Supplementary Figure 7) or text files. The python script was written to scan the text files for “CG” dinucleotides and quantify the number of CG versus TG dinucleotides per sample analyzed which finally provides methylation percentage. Several lollipop diagrams per gene were manually compared to the methylation percentage generated by the script to ensure accuracy of the generated methylation levels.

### Statistical tests

All plotted data were represented as mean + SEM. An unpaired student’s t-test was used to calculate significance and P < 0.05 was used as the significance cut-off for normally distributed data. For count-based data, a nonparametric Mann Whitney U test was used to estimate significance. Chi square analysis was carried out using a web based calculator (http://www.socscistatistics.com/tests/chisquare2/Default2.aspx) and a significance of P < 0.05 was used as a cutoff. All data were generated using GraphPad Prism version 6.00 for Windows, GraphPad Software, La Jolla California USA, www.graphpad.com.

## Additional Information

**How to cite this article**: Mahadevan, S. *et al*. Maternally expressed NLRP2 links the subcortical maternal complex (SCMC) to fertility, embryogenesis and epigenetic reprogramming. *Sci. Rep.* **7**, 44667; doi: 10.1038/srep44667 (2017).

**Publisher's note:** Springer Nature remains neutral with regard to jurisdictional claims in published maps and institutional affiliations.

## Supplementary Material

Supplementary Information

Supplementary video S1 and S2
