# Erratum: Maternally expressed NLRP2 links the subcortical maternal complex (SCMC) to fertility, embryogenesis and epigenetic reprogramming

**DOI:** 10.1038/srep46434

**Published:** 2017-04-19

**Authors:** Sangeetha Mahadevan, Varsha Sathappan, Budi Utama, Isabel Lorenzo, Khalied Kaskar, Ignatia B. Van den Veyver

Scientific Reports 7: Article number: 4466710.1038/srep44667; published online: 03 20 2017; updated: 04 19 2017

The original version of this Article contained an incorrect affiliation list. The correct affiliations are listed below:

Affiliation 1

Department of Obstetrics and Gynecology, Baylor College of Medicine, Houston, Texas, 77030, USA.

Sangeetha Mahadevan, Khalied Kaskar & Ignatia B. Van den Veyver

Affiliation 2

Jan and Duncan Neurological Research Institute, Texas Children’s Hospital, Houston, Texas, 77030, USA.

Sangeetha Mahadevan & Ignatia B. Van den Veyver

Affiliation 3

Century Scholars Program, Rice University, Houston, Texas, 77005, USA.

Varsha Sathappan

Affiliation 4

Shared Equipment Authority, Rice University, Houston, Texas, 77005, USA.

Budi Utama

Affiliation 5

Department of Molecular Human Genetics, Baylor College of Medicine, Houston, Texas, 77030, USA.

Isabel Lorenzo & Ignatia B. Van den Veyver

Affiliation 6

Interdepartmental Graduate Program in Translational Biology and Molecular Medicine, Baylor College of Medicine, Houston, Texas, 77030, USA.

Sangeetha Mahadevan

These errors have now been corrected in the PDF and HTML versions of this Article.

