# Supplementary material for: Maternally expressed NLRP2 links the subcortical maternal complex (SCMC) to fertility, embryogenesis and epigenetic reprogramming
Source: Sci Rep. 2017 Mar 20;7:44667. doi: 10.1038/srep44667 (PMC5357799; doi:10.1038/srep44667)
Supplement: Supplementary Information [file srep44667-s1.pdf]

Maternally expressed NLRP2 links the subcortical maternal complex (SCMC) to fertility, embryogenesis and epigenetic reprogramming.

Sangeetha Mahadevan[1,2,6], Varsha Sathappan[3], Budi Utama[4], Isabel Lorenzo[5], Khalied Kaskar[1], Ignatia B. Van den Veyver[1,2,5*]

[1]Department of Obstetrics and Gynecology; Baylor College of Medicine; Houston, Texas, 77030; USA

[2]Jan and Duncan Neurological Research Institute; Texas Children's Hospital; Houston, Texas, 77030; USA

[3]Century Scholars Program; Rice University; Houston, Texas, 77005; USA

[4]Shared Equipment Authority; Rice University; Houston, Texas, 77005; USA

[5]Department of Molecular Human Genetics; Baylor College of Medicine; Houston, Texas, 77030; USA

[6]Interdepartmental Graduate Program in Translational Biology and Molecular Medicine; Baylor College of Medicine; Houston, Texas, 77030; USA

[*]Correspondence: iveyver@bcm.edu

Supplemental Experimental Procedures:

Generation of the targeted ES cells

The HTGR06008_A_1_C01 targeting vector was obtained from the Knockout Mouse Project (KOMP) which was electroporated into mouse embryonic stem cells (JM8A3 line) at the Mouse Embryonic Stem (ES) Cell Core at Baylor College of Medicine. The targeting vector contained *Nlrp2* sequences from the C57NL/6N strain. The targeting construct and homologous recombination scheme is provided in Figure 1A. Southern Blotting was performed using enzymes EcoRV (5′-arm) and NheI (3′-arm) on the electroporated ES cells to select for clones that were positive from both 5′ and 3′ ends. Digestion with EcoRV followed by probing with the 5' probe results in a 16.8 kb wild type and 11.8 kb targeted fragment. Similarly, digestion with NheI followed by probing with the 3' probe results in a 15.4 kb targeted and 9.4 kb wild type fragment (Figure S1B). Clones which contained targeted alleles on both 5' and 3' arms were expanded for confirmation. ES cells from expanded recombinant clones 3F1 and 3F6 were microinjected into C57BL/6J-Tyrc-2J blastocysts. Ninety-six blastocysts were then microinjected into eight pseudopregnant females at the Genetically Engineered Mouse Core at Baylor College of Medicine. Clone 3F1 yielded 10 (9 males, 1 female) chimeric offspring and 3F6 yielded 7 (5 males, 2 females) chimeric offspring. All male chimeras were bred at 7 weeks of age with age matched C57BL/6J-Tyrc-2J females. Of the 17 male chimeras bred, a single chimera (derived from the 3F1 clone) sired germ line transmitted. $Nlrp2^{tm1a/+}$ mice were bred to generate $Nlrp2^{+/+}$, $Nlrp2^{tm1a/+}$ *and* $Nlrp2^{tm1a/tm1a}$ offspring. Genotyping was performed by PCR on tail DNA by standard methods. Primer sequences are provided in supplementary table 3. The wild type allele yields a product of 140 bp while the tm1a allele yields a product of 190 bp.

Gene expression studies

Whole brain, liver, spleen, heart, lung, kidney, uterus and ovaries were dissected free of surrounding fat and blood in 1X phosphate buffered saline (PBS), flash frozen in liquid nitrogen and stored in -80 °C. Embryonic day 9.5 (E9.5) embryos were dissected free of maternal decidua and washed multiple times in PBS prior to being flash frozen. Five $Nlrp2^{M-/Z+}$ and $Nlrp2^{M+/Z+}$ embryos were used for gene expression analysis. The tissues were homogenized in Qiazol using a motorized pestle. RNA was isolated from the tissues using the Qiagen miRNeasy kit (Qiagen, Cat # 217004). 500ng of RNA was reverse transcribed to cDNA using the qScript cDNA SuperMix (Quanta Biosciences, Cat# 95048-500. Quantitative RT-PCR was conducted using the PerfeCTa SYBR Green FastMix Reaction Mix (Quanta Biosciences, Cat# 95072-250). Primer sequences used to carry out qRT-PCR are provided in Supplementary table 2. Gene expression within the ovary was normalized to *Gapdh* and germ cell specific Mouse vasa homolog (*Mvh*) while for the other tissues, and E9.5 embryos; gene expression was normalized only to *Gapdh*.

Timed mating

Seven to eight-week old female $Nlrp2^{+/+}$ and $Nlrp2^{tm1a/tm1a}$ were housed with >8 week old $Nlrp2^{+/+}$ male mice between 1600 – 1800 hours and checked for the presence of vaginal plug the following morning. If a vaginal plug was observed, the male was removed from the cage and females were euthanized on E9.5. Embryos were photographed and frozen at -20 °C for DNA isolation and bisulfite sequencing studies.

Superovulation

Three to five-week old mice were superovulated by intraperitoneal injections of 5U of pregnant mare serum gonadotropin (PMSG) followed by 5U of human chorionic gonadotropin (hCG) 47 hours later. A proven male was then introduced in the cage for in vivo fertilization. Mice were inspected the following morning for the presence of a copulatory plug and sacrificed approximately 22 hours after the hCG injection. After humane euthanasia, serum was collected by cardiac puncture to assay for steroid hormone levels. Cumulus oocyte complexes (COCs) were recovered from the ampulla and the oocyte was dissociated from the cumulus by mechanical pipetting in 3mg/ml hyaluronidase (Sigma-Aldrich, Cat # H4272-30MG).

Serum levels of steroid hormones were assayed at the University of Virginia, Center for Research in Reproduction, Ligand Assay and Analysis Core. The following hormones were assayed: Anti-Mullerian Hormone (AMH) by ELISA, Estradiol by ELISA, Follicle stimulating hormone (FSH) and Luteinizing hormone (LH) by multiplex ELISA and Progesterone by ELISA.

Histological studies

For histological studies, ovaries were collected from mice at various time points by dissecting in 1X PBS and fixed in Formalde-Fresh (Fisher Scientific, Pittsburg, PA) overnight at 4 °C. Fixed tissues were washed in 1X PBS twice for 45 minutes and subsequently dehydrated for an hour in 50% and 70% ethanol. They were further dehydrated for a total of 3 changes of 100% ethanol for an hour each at room temperature. The tissues were then treated with xylene for 30 minutes each for a total of 3 changes followed by 2 changes of paraffin for an hour each at room temperature prior to embedding tissues in paraffin. Paraffin embedded tissues were then serially sectioned at 5-8 μM thicknesses and used for hematoxylin and eosin staining (H&E), immunohistochemistry, immunofluorescence and TUNEL assays. H&E stained sections were observed by microscopy for gross oocyte morphology. Every 10th section was photographed and oocytes within each section were counted, including distribution of various follicle stages. Oocytes were described as being unhealthy/degrading if they exhibited any of the following histological signs: abnormal oocyte morphology, fragmented oocyte appearance, collapsed zona pellucida, increased number of pyknotic granulosa cells and abnormal granulosa cell proliferation. 3-4 *Nlrp2*$^{+/+}$ and *Nlrp2*$^{tm1a/tm1a}$ females were used for analysis.

LacZ staining

Tissues from 3-week-old mice were collected in 1X PBS and fixed using β-Gal fixative solution (0.5% glutaraldehyde, 2.0% paraformaldehyde, 2 mM MgCl2, 1.25 mM EGTA and 1X PBS) for 30 minutes. The tissues were washed thrice in 1X PBS for 15 minutes each. The tissues were then stained in dark for 1-6 hours at 37 °C in a staining solution (1X PBS, 2 mM MgCl2, 2 mL 1M MgCl2, 5 mM potassium ferrocyanide, 5 mM potassium ferricyanide and 1 mg/mL X-Gal solution). The tissues were then rinsed thrice in 1X PBS for 10 minutes each. The tissues were then fixed in formalin for 10 minutes followed by rinsing in 1X PBS thrice for 5 minutes each. If the tissues were to be sectioned and counterstained, they were processed like the histological analyses as described above and counterstained with nuclear fast red stain. If the tissues were to be imaged as whole mounts, they were cleared in a glycerol/potassium hydroxide series as follows – 30% glycerol/1% potassium hydroxide for 3 days at 37 °C followed by 50% glycerol/1% potassium hydroxide for 3 days at 37 °C followed by 80% glycerol/1% potassium hydroxide for 3 days at 37 °C followed by 100% glycerol for 3 days at 37 °C. These cleared tissues were then imaged.

**Null female**	**Litter 1**	**Litter 2**	**Litter 3**	**Litter 4**	**Litter 5**
1	No pups	2 pups delivered E23 – both deceased at birth, gross morphological defects	No pups	2 pups delivered E23 – both deceased at birth, gross morphological defects	1 pup delivered E19 1.8g at birth and gained 0.1g by P10. Deceased P16
2	No pups	No pups	No pups	No pups	Did not plug, >3 males tested
3	No pups	No pups	No pups	No pups	Did not plug, >3 males tested
4	6 apparently normal pups delivered E20	4 pups delivered E19 – normal body weight at birth, dramatic doubling in body weight by P10	3 pups delivered E20 – deceased at birth, gross morphological defects	1 grossly abnormal deceased and 3 apparently normal pups delivered E20	Did not plug, >3 males tested
5	9 apparently normal pups delivered E20	No pups	5 apparently normal pups delivered E20 – 4 pups deceased by P1	Did not plug, >3 males tested	
6	No pups	9 apparently normal pups delivered E18	8 apparently normal pups delivered E18	Did not plug, >3 males tested	
7	5 apparently normal pups delivered E19	8 apparently normal pups delivered E22	5 apparently normal pups delivered E19		
8	4 apparently normal pups delivered E19	No pups	2 pups delivered E20 – one deceased at birth with gross morphological defects, other deceased P1		
9	2 pups delivered E22 – both deceased at birth, gross morphological defects				

Supplementary table 1: Reproductive outcomes for 9 individual *Nlrp2*$^{tm1a/tm1a}$ females across various litters. In all instances, females were bred with *Nlrp2*$^{+/+}$ males.

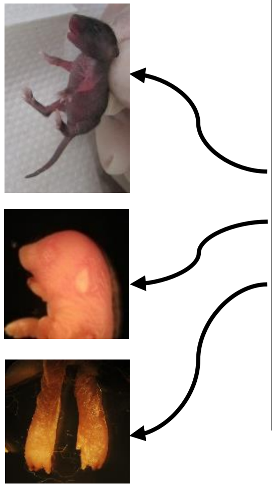

Pup	***Genotype***	***Cdkn1c***	***Impact***	***Nesp***	***Zac1***	***Kncq1ot1***
**1**	$Nlrp2^{M+/Z+}$	66	NA	66	50	47
**2**	$Nlrp2^{M+/Z+}$	48	14	70	54	64
**3**	$Nlrp2^{M+/Z+}$	47	4	75	64	61
**4**	$Nlrp2^{M+/Z+}$	41	10	73	64	67
**5**	$Nlrp2^{M-/Z+}$	27	57	54	74	60
**6**	$Nlrp2^{M-/Z+}$	8	80	81	86	42
**7**	$Nlrp2^{M-/Z+}$	NA	90	48	100	64
**8**	$Nlrp2^{M-/Z+}$	1	18	18	NA	97
**9**	$Nlrp2^{M-/Z+}$	57	52	73	96	75
**10**	$Nlrp2^{M-/Z+}$	37	54	94	51	33
**11**	$Nlrp2^{M-/Z+}$	NA	100	53	NA	0
**12**	$Nlrp2^{M-/Z+}$	NA	100	79	94	86
**13**	$Nlrp2^{M-/Z+}$	67	99	78	72	92

Supplementary table 2A: Methylation levels of individual pups used in the bisulfite cloning data in figure 5A. Pictures of pup #9, 10 and 11 from figure 2 are provided as they correspond with the analysis presented in this table.

Embryo	*Genotype*	*Cdkn1c*	*Impact*	*Nesp*	*Zac1*	*Kncq1ot1*	*Snrpn*	*Peg3*	*Igf2r*	*H19*	*Gtl2*	*Mest*
**1**	$Nlrp2^{M+/Z+}$	25	72	74	61	28	19	59	48	39	NA	NA
**2**	$Nlrp2^{M+/Z+}$	10	44	73	57	35	25	73	36	70	NA	NA
**3**	$Nlrp2^{M+/Z+}$	20	61	71	57	28	56	54	52	2	NA	NA
**4**	$Nlrp2^{M+/Z+}$	9	38	59	63	34	31	57	56	21	NA	NA
**5**	$Nlrp2^{M+/Z+}$	NA	NA	NA	NA	NA	NA	NA	NA	NA	60	NA
**6**	$Nlrp2^{M+/Z+}$	NA	NA	NA	NA	NA	NA	NA	NA	NA	NA	38
**7**	$Nlrp2^{M+/Z+}$	NA	NA	NA	NA	NA	NA	NA	NA	NA	31	44
**8**	$Nlrp2^{M+/Z+}$	NA	NA	NA	NA	NA	NA	NA	NA	NA	60	38
**9**	$Nlrp2^{M+/Z+}$	NA	NA	NA	NA	NA	NA	NA	NA	NA	53	50
**10**	$Nlrp2^{M-/Z+}$	15	57	63	91	29	19	73	57	28	40	0
**11**	$Nlrp2^{M-/Z+}$	14	51	38	68	36	38	85	50	26	19	50
**12**	$Nlrp2^{M-/Z+}$	27	40	74	68	56	47	75	63	55	63	40
**13**	$Nlrp2^{M-/Z+}$	36	50	59	74	40	46	55	70	57	13	63
**14**	$Nlrp2^{M-/Z+}$	NA	NA	NA	NA	NA	NA	NA	NA	NA	44	57
**15**	$Nlrp2^{M-/Z+}$	NA	NA	NA	NA	NA	NA	NA	NA	NA	46	33

Supplementary table 2B: Methylation levels of individual embryos used in the bisulfite cloning data in figure 5B. Samples which were not analyzed are referred to as NA.

Gene name	Objective	Forward	Reverse	Tm	Reference
Nlrp2	Genotyping	ATTAAGGACGCCAGCCTGAG (P3)	GGGCAGAGAGAGGAGGGGTAT (P4)	60˚C	Present
Nlrp2	qRT-PCR	AACACTGAGCCTGAAACACTTGGA	CAGTTCAGTGGAGTGATGGAGCA	60˚C	Present
Gapdh	qRT-PCR	TCACCACCATGGAGAAGGC	GCTAAGCAGTTGGTGGTGCA	60˚C	Present
Mvh	qRT-PCR	CCAAAAGTGACATATATACCC	TTGGTTGATCAGTTCTCGAG	60˚C	(Nitta et al., 2013)
Zac1	qRT-PCR	TTTTCTTTGCCTAGCTTAACCTACTACTT	CACAATCCTCTTGGGATACAAACTAA	60˚C	(Varrault et al., 2006)
Grb10	qRT-PCR	AGGATCATCAAGCAACAAGGTCTC	ATTACTCTGGCTGTCACGAAGGA	60˚C	
Mest	qRT-PCR	CAACAATGACGGCAACCTGGT	TCTGAATTTCTTCCTTTGATTAATGTACTGTA	60˚C	
Impact	qRT-PCR	ACCGAGCCAGACCCAGATG	TGAGGTCCAATGTTTTAACTG	60˚C	Present
Snrpn	qRT-PCR	TGCTCGTGTTGCTGCTACTG	GCAGTAAGAGGGGTCAAAAGC	60˚C	(Ciccone et al., 2009)
Igf2r	qRT-PCR	GCACAGAATCCAGACTAGCATTACA	CCTCCTTATCAGCTTTAAATATGTCTTTCTT	60˚C	(Varrault et al., 2006)
Cdkn1c	qRT-PCR	AACGTCTGAGATGAGTTAGTTTAGAGG	AAGCCCAGAGTTCTTCCATCGT	60˚C	
Peg3	qRT-PCR	TTGGACTGGACAGAGATGATGACA	ATTCTGGTATGACTCGGCATCCT	60˚C	
Zac1	Bisulfite sequencing	GGGTAGGTAAGTAGTGATAA (outer)	CCTAAAACACCAAAATAACA (outer)	55.7˚C	(Hiura et al., 2006)
		ATTTGGGTGTTTTAGTTGTA (inner)	TACAAAACCAAAACCCTTAC (inner)	55.7˚C	
Impact	Bisulfite sequencing	AGTAGGTTTTGTTGTATTGT (outer)	ATATAACAACTCAACCAAAC (outer)	50.4˚C	(Hiura et al., 2006)
		TTTTTTAATATGAGTAGGTT (inner)	ATAACTCAACAACCAAAC (inner)	50.4˚C	
Cdkn1c	Bisulfite sequencing	AGGATTTAGTTGGTAGTAGT (outer)	TATCCTATCCAACTTAAACC (outer)	52.6˚C	(Fan et al., 2005)
		AGGATTTAGTTGGTAGTAGT (inner)	TTTTCAATTTCAACAACACC (inner)	52.6˚C	
Nesp	Bisulfite sequencing	GTAATTTTATAGGGTTTTATTG (outer)	ATCCATTCTCTTAAATACTCACC (outer)	55.7˚C	(Liu et al., 2000)
		GAGAGGATTAGTGGAGGTATTTTT (inner)	ACTCACCCTCTAACTCTACAAAAAAT (inner)	55.7˚C	
Kcnq1ot1	Bisulfite sequencing	TAAGGTGAGTGGTTTAGGAT (outer)	AATCCCCCACACCTAAATTC (outer)	59.3˚C	(Hiura et al., 2006)
		TAAGGTGAGTGGTTTAGGAT (inner)	CCACTATAAACCCACACATA (inner)	59.3˚C	
Snrpn	Bisulfite sequencing	TATGTAATATGATATAGTTTAGAAATTAG (outer)	AATAAACCCAAATCTAAAATATTTTAATC (outer)	52.6˚C	(Market-Velker et al., 2010)
		AATTTGTGTGATGTTTGTAATTATTTGG (inner)	ATAAAATACACTTTCACTACTAAAATCC (inner)	52.6˚C	
Peg3	Bisulfite sequencing	TTTTGATAAGGAGGTGTTT (outer)	ACTCTAATATCCACTATAATAA (outer)	50˚C	(Market-Velker et al., 2010)
		AGTGTGGGTGTATTAGATT (inner)	TAACAAAACTTCTACATCATC (inner)	53˚C	
Igf2r	Bisulfite sequencing	GGGGAATTGAGGTAAGTTAGGGTTTT	TCTTATAACCCAAAAATCTTCACCCTAAC	55˚C	(Tomizawa et al., 2011)
H19	Bisulfite sequencing	GAGTATTTAGGAGGTATAAGAATT (outer)	ATCAAAAACTAACATAAACCTCT (outer)	52.6˚C	(Market-Velker et al., 2010)
		GTAAGGAGATTATGTTTATTTTTGG (inner)	CCTCATTAATCCCATAACTAT (inner)	52.6˚C	
Gtl2	Bisulfite sequencing	TGGGTAAGTTTTATGGTTTATTGTATATAATGTTG	ATCCCCTATACTCAAAACATTCTCCATTAAC	60˚C	Present
Mest	Bisulfite sequencing	GYGGGGTTTAGTGGGTTTTAAAAG	CACRAACTAACAACACCCACCTAC	60˚C	Present

Supplementary table 3: Primer sequences for primers used in the present study; all primers designed in this study were done so in the NCBI37/mm9 assembly.

Supplementary video figure legends

Supplementary video S1

Nlrp2$^{+/+}$ females were superovulated and mated with *Nlrp2*$^{+/+}$ males. Zygotes were recovered from the ampulla, denuded and placed in the EmbryoScope time-lapse imaging system for ~5 days. A media change was performed roughly on day 3 causing the embryo positions to change. Embryoscopy videos have been compressed to 3 hours/second.

Supplementary video S2

Nlrp2$^{tm1a/tm1a}$ females were superovulated and mated with *Nlrp2*$^{+/+}$ males. Zygotes were recovered from the ampulla, denuded and placed in the EmbryoScope time-lapse imaging system for ~5 days. A media change was performed roughly on day 3 causing the embryo positions to change. Embryoscopy videos have been compressed to 3 hours/second.

Supplementary references

Ciccone, D. N., Su, H., Hevi, S., Gay, F., Lei, H., Bajko, J., Xu, G., Li, E. & Chen, T. 2009. KDM1B is a histone H3K4 demethylase required to establish maternal genomic imprints. *Nature,* 461**,** 415-8.
Fan, T., Hagan, J. P., Kozlov, S. V., Stewart, C. L. & Muegge, K. 2005. Lsh controls silencing of the imprinted Cdkn1c gene. *Development,* 132**,** 635-44.
Hiura, H., Obata, Y., Komiyama, J., Shirai, M. & Kono, T. 2006. Oocyte growth-dependent progression of maternal imprinting in mice. *Genes Cells,* 11**,** 353-61.
Liu, J., Yu, S., Litman, D., Chen, W. & Weinstein, L. S. 2000. Identification of a methylation imprint mark within the mouse Gnas locus. *Mol Cell Biol,* 20**,** 5808-17.
Market-Velker, B. A., Zhang, L., Magri, L. S., Bonvissuto, A. C. & Mann, M. R. 2010. Dual effects of superovulation: loss of maternal and paternal imprinted methylation in a dose-dependent manner. *Hum Mol Genet,* 19**,** 36-51.
Nitta, M., Imamura, M., Inoue, Y., Kunitomo, Y., Lin, Z. Y., Ogawa, T., Yogo, K., Ishida-Kitagawa, N., Fukunaga, N., Okano, H., Sato, E., Takeya, T. & Miyoshi, J. 2013. Aberrant gene expression and sexually incompatible genomic imprinting in oocytes derived from XY mouse embryonic stem cells in vitro. *PLoS One,* 8**,** e58555.
Tomizawa, S., Kobayashi, H., Watanabe, T., Andrews, S., Hata, K., Kelsey, G. & Sasaki, H. 2011. Dynamic stage-specific changes in imprinted differentially methylated regions during early mammalian development and prevalence of non-CpG methylation in oocytes. *Development,* 138**,** 811-20.
Varrault, A., Gueydan, C., Delalbre, A., Bellmann, A., Houssami, S., Aknin, C., Severac, D., Chotard, L., Kahli, M., Le Digarcher, A., Pavlidis, P. & Journot, L. 2006. Zac1 regulates an imprinted gene network critically involved in the control of embryonic growth. *Dev Cell,* 11**,** 711-22.

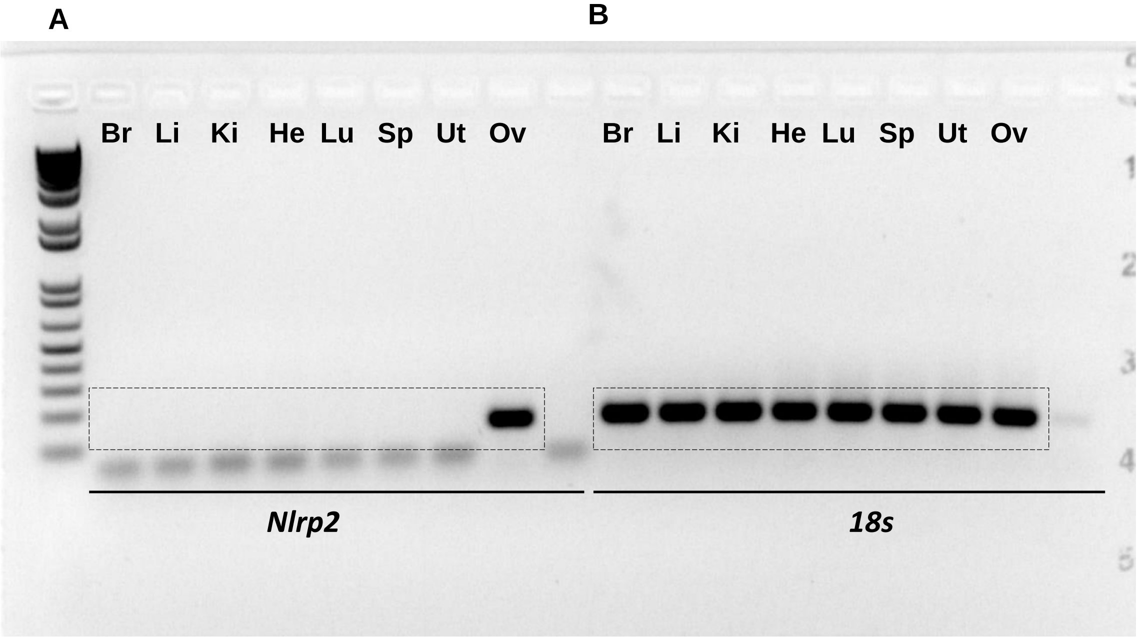

Supplementary figure 1: *Nlrp2* transcript is only detected in the ovary

(A) RT-PCR of *Nlrp2* in the brain (br), liver (li), kidney (ki), heart (he), lung(lu), spleen(sp), uterus(ut) and ovary (ov) results in a positive band only in the ovary. Cropped images in Figure 1A are marked by dashed black boxes.

(B) RT-PCR of *Gapdh* in the brain (br), liver (li), kidney (ki), heart (he), lung(lu), spleen(sp), uterus(ut) and ovary (ov) serves as a control for the RT-PCR reaction. Cropped images in Figure 1A are marked by dashed black boxes.

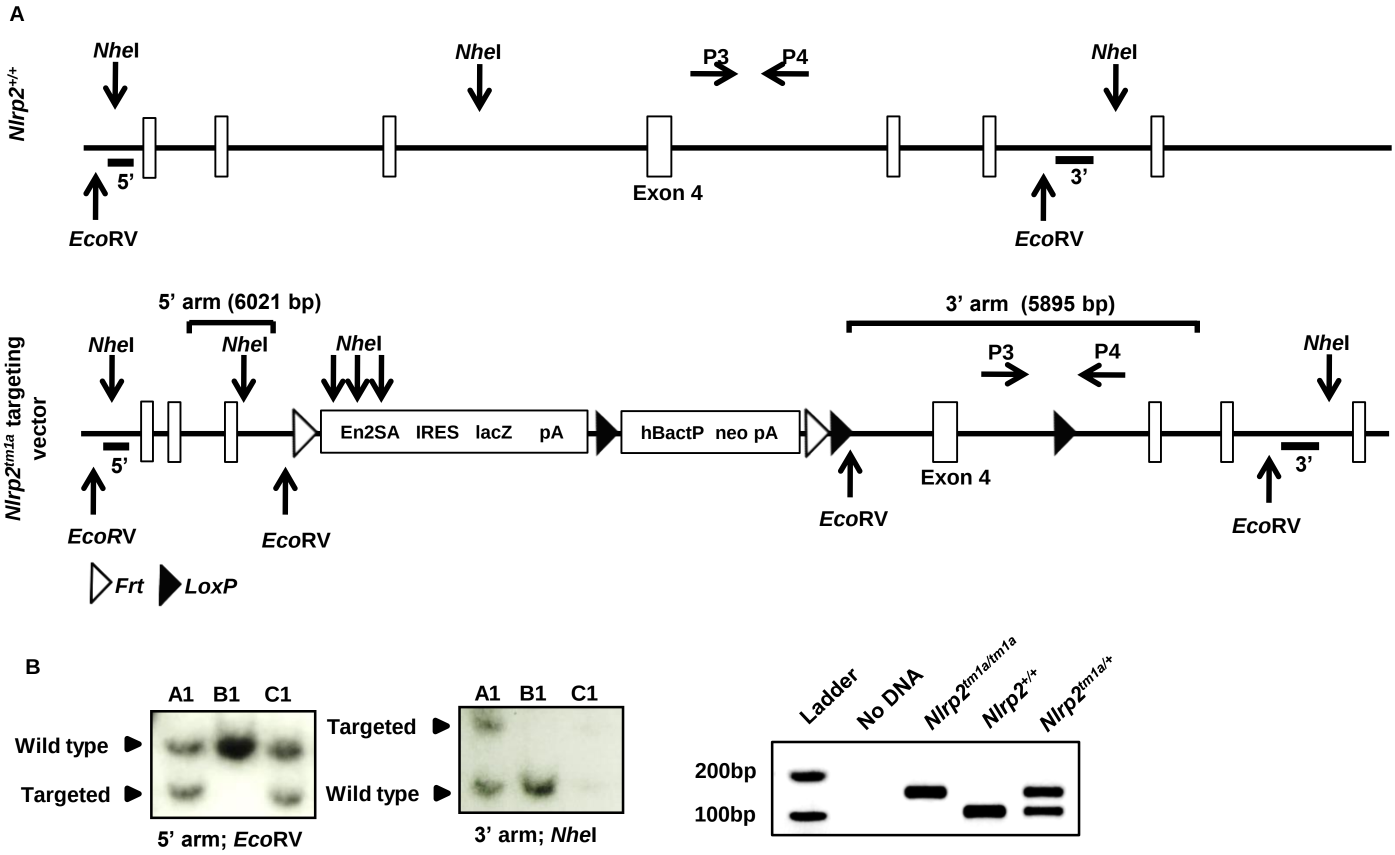

Supplementary figure 2: Generation of *Nlrp2* targeted mice

A schematic of the targeting vector used to generate *Nlrp2*$^{tm1a/tm1a}$ mice. The targeting construct was inserted between exons 3 and 4 by homologous recombination. (B) Southern hybridization of *EcoR*V-digested genomic DNA from ES-cells or targeted mice with the 5' probe was used to distinguish the 11.8 kb recombined fragment from the 16.8 kb wild type fragment at the 3' arm. Southern hybridization of *Nhe*I-digested genomic DNA with the 3' probe was used to distinguish the 15.4 kb recombined fragment from the 9.4 kb wild type fragment at the 3'arm. PCR-based genotyping used to detect wild type, heterozygous and homozygous mutant mice was carried out on genomic DNA from tail snips using primers P3 and P4, which generate a 140 bp product from the wild-type allele and a 190 bp product from the targeted allele due to presence of a *lox*P element.

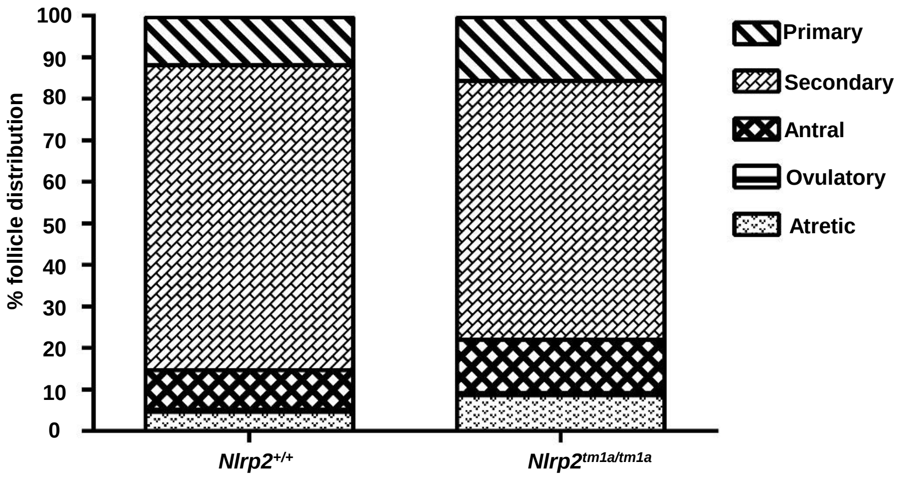

Supplementary figure 3: Follicle distribution in ovaries from *Nlrp2$^{tm1a/tm1a}$* mice compared to *Nlrp2$^{+/+}$* mice
Hematoxylin and eosin staining was carried out on ovaries retrieved at P21 (n=3-4) to assess follicular maturation dynamics. Distribution of follicle classes, represented as percentages of the total follicular counts, reveals no difference in the abundance of primary, antral or ovulatory follicles, but a higher percentage of atretic and lower percentage of secondary follicles.

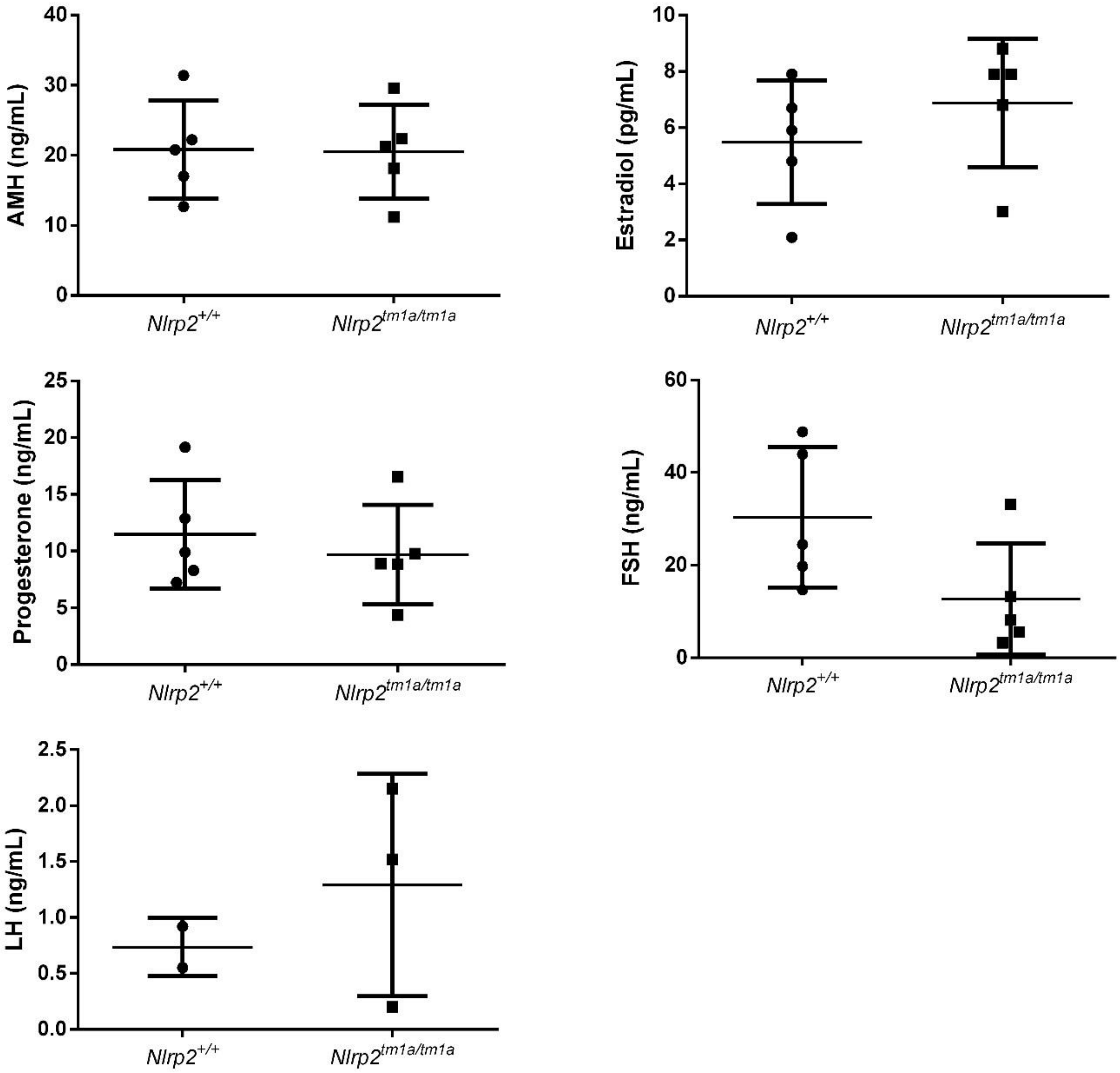

Supplementary figure 4: Analysis of steroid hormones indicates no difference in neuroendocrine signaling in *Nlrp2*$^{tm1a/tm1a}$ females compared to *Nlrp2*$^{+/+}$ females

Females of both genotypes (n=3-5) were superovulated and serum was collected to assay for steroid hormone levels. No significant differences were detected in serum levels of Anti-Mullerian Hormone (AMH), Estradiol, Progesterone, Follicle Stimulating Hormone (FSH) and Luteinizing Hormone (LH).

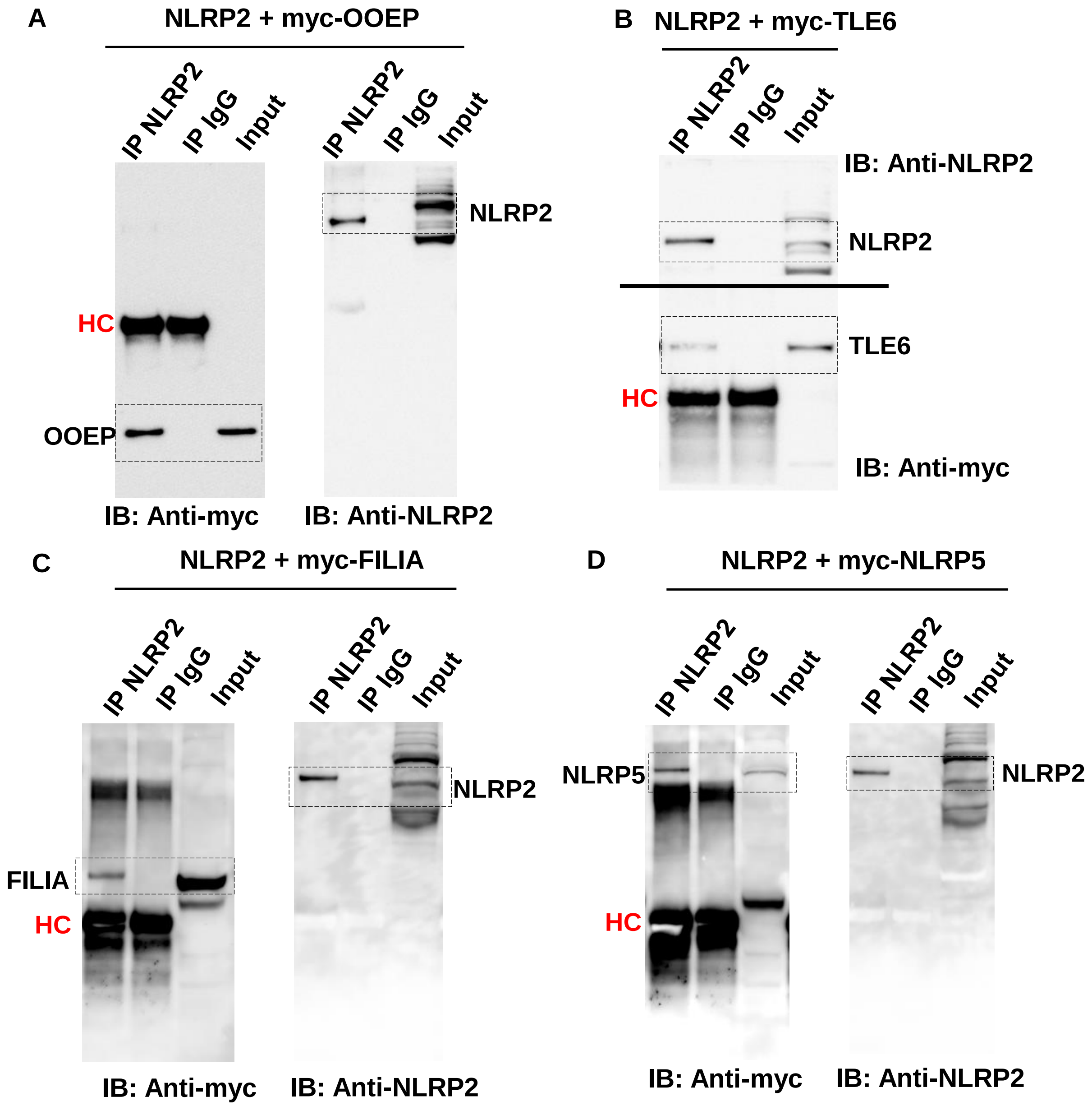

Supplementary figure 5: NLRP2 interacts with SCMC proteins, OOEP, TLE6, FILIA and NLRP5

(A) Co-transfection of NLRP2 and myc-OOEP followed by immunoprecipitation by NLRP2 reveals an interaction between NLRP2 and OOEP as evidenced by the presence of a band ~19kDa in the IP and Input lane when probed with Anti-myc. Heavy chain fragments in the IP lanes are marked by "HC" in red. The blot was stripped and probed with Anti-NLRP2 and presence of NLRP2 in the IP and input lane confirms the immunoprecipitating protein was present. Cropped sections in Figure 4A are marked by dashed boxes.

(B) Co-transfection of NLRP2 and myc-TLE6 followed by immunoprecipitation by NLRP2 reveals an interaction between NLRP2 and TLE6 as evidenced by the presence of a band ~60kDa in the IP and Input lane when probed with Anti-myc. The top fragment of the blot (>100kDa) was probed using Anti-NLRP2 and presence of NLRP2 in the IP and input lane confirms the immunoprecipitating protein was present. The region where the blot was cut to be probed with anti-myc and anti-NLRP2 is marked with a black straight line. Heavy chain fragments in the IP lanes are marked by "HC" in red. Cropped sections in Figure 4A are marked by dashed boxes.

(C) Co-transfection of NLRP2 and myc-FILIA followed by immunoprecipitation by NLRP2 reveals an interaction between NLRP2 and FILIA as evidenced by the presence of a band in the IP and Input lane when probed with Anti-myc. Heavy chain fragments in the IP lanes are marked by "HC" in red. The blot was stripped and probed with Anti-NLRP2 and presence of NLRP2 in the IP and input lane confirms the immunoprecipitating protein was present. Cropped sections in Figure 4A are marked by dashed boxes.

(D) Co-transfection of NLRP2 and myc-NLRP5 followed by immunoprecipitation by NLRP2 reveals an interaction between NLRP2 and NLRP5 as evidenced by the presence of a band in the IP and Input lane when probed with Anti-myc. Heavy chain fragments in the IP lanes are marked by "HC" in red. The blot was stripped and probed with Anti-NLRP2 and presence of NLRP2 in the IP and input lane confirms the immunoprecipitating protein was present. Cropped sections in Figure 4A are marked by dashed boxes.

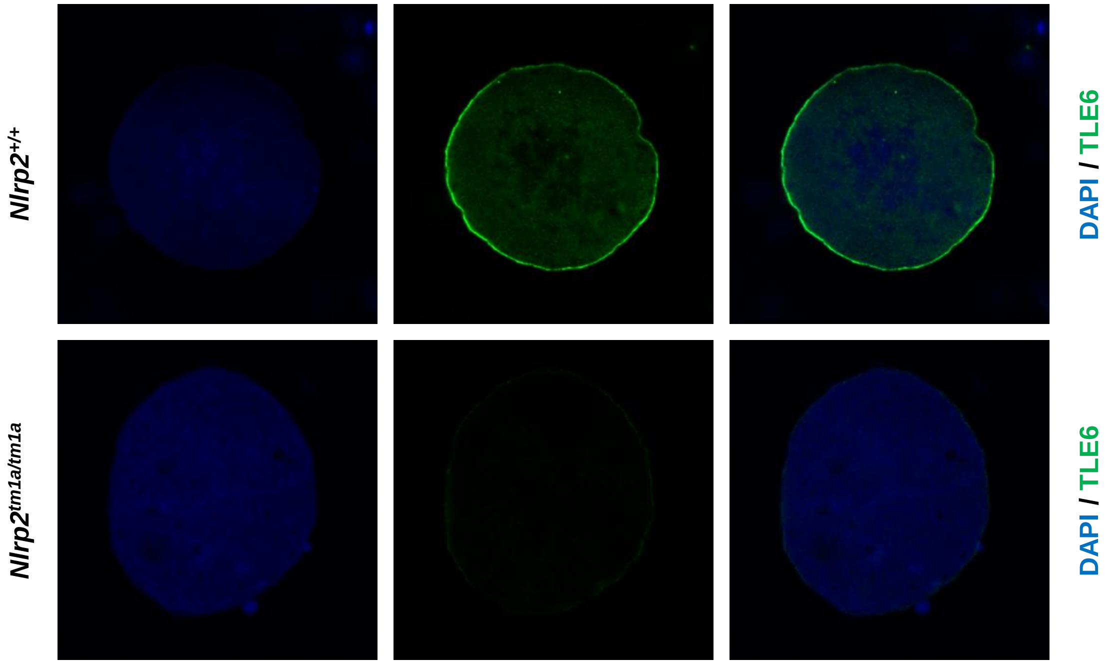

Supplementary figure 6: Assessing if TLE6 staining differs depending on processing conditions. Immunofluorescent staining of paraffin embedded oocytes derived from control and *Nlrp2*$^{tm1a/tm1a}$ females processed in primary antibody for 1 hour at room temperature. Contrary to published reports, no difference in control staining is noted when treated with primary antibody for 1 hour at room temperature when compared to overnight at 4˚C (Figure 4C). Unlike staining pattern observed in Figure 4C, TLE6 staining in oocytes from *Nlrp2*$^{tm1a/tm1a}$ dams are almost absent.
